# Field‐Frustrated Cooperative Distortions: Suppressing Jahn‐Teller Ordering via Microwave Annealing

**DOI:** 10.1002/advs.76001

**Published:** 2026-06-04

**Authors:** Daryoosh Vashaee, Kelvin Dsouza

**Affiliations:** ^1^ Electrical and Computer Engineering Department North Carolina State University Raleigh North Carolina USA; ^2^ Materials Science and Engineering Department North Carolina State University Raleigh North Carolina USA

**Keywords:** cooperative Jahn‐Teller distortion, metastable phases, microwave annealing, phonon‐field interaction, spinel oxides

## Abstract

Cooperative Jahn‐Teller (CJT) distortions in correlated oxides couple local electronic degeneracy to long‐range lattice symmetry breaking, giving rise to functional properties such as orbital ordering and magnetoelastic effects. In copper ferrite (CuFe_2_O_4_), this coupling drives a well‐known cubic‐to‐tetragonal phase transition under equilibrium thermal processing. Here, we investigate how microwave (MW) annealing modifies oxidation, local structure, and global symmetry in CuFe_2_O_4_ using synchrotron X‐ray diffraction, pair distribution function analysis, and X‐ray photoelectron spectroscopy. We find that MW processing results in complete oxidation to Cu^2+^ and restoration of short‐range oxygen polyhedral order, yet the crystal structure remains cubic, in contrast to furnace‐annealed samples that undergo CJT‐driven symmetry lowering. Notably, three samples with identical cubic symmetry, nanocrystalline, MW‐annealed nanocrystalline, and samples relaxed from a tetragonal CJT state under MW exposure, exhibit distinct Cu 2p satellite intensities and binding energies, indicating different local electronic environments. These results demonstrate that MW annealing decouples local structural relaxation from long‐range cooperative ordering. Using a custom‐built pulsed MW system with spatially resolved infrared thermography, we show that this behavior cannot be attributed to rapid quenching. The findings establish MW fields as a pathway to stabilize symmetry‐frustrated states in complex oxides.

## Graphical abstract

Microwave annealing decouples local Jahn‐Teller distortions from long‐range cooperative ordering in CuFe_2_O_4_, stabilizing a metastable cubic phase that remains locally distorted yet globally symmetry‐frustrated. Synchrotron XRD, PDF, XPS, and in situ thermal cycling reveal how non‐equilibrium MW‐phonon interactions suppress cooperative orbital‐lattice coherence while preserving short‐range oxygen coordination, offering a pathway to engineer field‐stabilized states in correlated oxides.

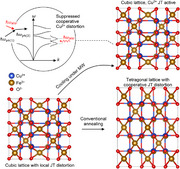

## 1 Introduction

Structural distortions in crystalline solids are often driven by the interplay between electronic degeneracies and lattice symmetry. In correlated oxides, where strong electron‐lattice interactions and orbital degrees of freedom play a central role, these distortions can become cooperative, meaning they do not occur as isolated local events but rather propagate coherently through the lattice. Among the most prominent of such distortions is the cooperative Jahn‐Teller distortion (CJT), which arises when Jahn‐Teller active ions, typically with partially filled *e_g_* orbitals (e.g., Cu^2+^, Mn^3+^), occupy symmetry‐equivalent sites in a periodic lattice [1, 2, 3].

Unlike a simple local Jahn‐Teller effect, which results in the distortion of an individual octahedron to remove orbital degeneracy, CJT distortions involve long‐range orbital‐lattice coupling that breaks the global symmetry of the crystal. This collective behavior leads to structural phase transitions, typically from high‐symmetry (cubic or rhombohedral) to lower‐symmetry (tetragonal, orthorhombic) phases. These transitions are energetically favored when the gain in electronic energy from lifting degeneracy outweighs the entropy and elastic penalties of distortion [4]. The resulting cooperative phases often exhibit rich physical properties, including orbital ordering, colossal magnetoresistance, multiferroicity, and strong magnetoelastic coupling [4, 5, 6, 7].

CJT transitions are particularly sensitive to perturbations such as pressure, strain, and electromagnetic fields because they depend on the long‐range coherence of local structural distortions. Any mechanism that introduces disorder, alters phonon lifetimes, or disrupts symmetry propagation can suppress or fundamentally alter the nature of the transition. This sensitivity makes CJT systems an ideal platform for probing how external energy inputs affect the formation of collective lattice states.

Among various external stimuli, MW fields offer a particularly intriguing form of non‐equilibrium perturbation. Unlike conventional heating, which progresses from the surface inward, MW irradiation couples directly to the dielectric properties of a material, enabling volumetric and often selective energy deposition through the oscillation of polar species or charge carriers [8]. This creates non‐uniform thermal environments characterized by localized hot spots, steep temperature gradients, and rapid ionic mobility [9, 10, 11, 12]. These effects have enabled accelerated synthesis and phase formation in a range of material systems, including ferrites and perovskites [13, 14], but their influence on symmetry‐breaking cooperative distortions remains largely unexplored.

Microwave‐assisted heating has also demonstrated effects well beyond conventional thermal processing, including low‐temperature sintering [15], de‐crystallization [16], enhancement of reaction rates [17], accelerated diffusion [18], and catalytic transformations in both organic and inorganic systems [19, 20]. These results suggest that MW irradiation can alter not only the rate but also the pathway of structural and chemical transformations, a behavior especially relevant in systems governed by delicate energy balances between local and collective distortions.

Beyond thermal effects, MW fields may also drive non‐thermal lattice responses. The oscillating electromagnetic field can induce polarization fluctuations, enhance non‐equilibrium ion transport, or even modulate phonon populations by coupling to lattice vibrational modes. In strongly correlated oxides, such as Jahn‐Teller‐active systems, these effects can alter both the dynamics and symmetry of atomic displacements, potentially disrupting the propagation of cooperative distortions even when local symmetry‐breaking units remain active [21, 22]. Mechanistic models suggest that MW energy couples to low‐frequency elastic lattice modes, producing non‐thermal phonon distributions and anomalously efficient oxygen diffusion [21], while recent experimental reviews document field‐driven enhancements in activation rates, polarization alignment, and diffusion kinetics even absent a bulk temperature gradient [22]. These non‐thermal effects may be responsible for disrupting long‐range Jahn‐Teller coherence in CuFe_2_O_4_ despite local oxidation and polyhedral ordering.

In this context, MW fields act not simply as a heat source but as a non‐equilibrium stimulus capable of promoting local relaxation while frustrating long‐range orbital‐lattice coupling. This raises the possibility of accessing metastable or symmetry‐frustrated states that are stabilized by interference with collective ordering mechanisms. Understanding how MW fields affect such symmetry‐breaking phenomena is thus essential for both fundamental insight and the rational design of functional oxide materials.

Copper ferrite (CuFe_2_O_4_) offers a well‐characterized and structurally accessible platform for studying CJT distortions in complex oxides. As a member of the spinel family (general formula AB_2_O_4_), CuFe_2_O_4_ crystallizes in a framework composed of a face‐centered cubic oxygen lattice, with cations occupying tetrahedral (A) and octahedral (B) interstices. It adopts an inverse spinel configuration, where Cu^2+^ predominantly occupies the octahedral B‐sites, while Fe^3+^ is distributed across both A and B sites [23, 24].

At high temperatures, CuFe_2_O_4_ stabilizes in a cubic spinel structure (space group $\mathrm{Fd}\bar{3}m$), but upon cooling, it undergoes a well‐known first‐order phase transition to a tetragonal structure (space group *I*4_1_/*amd*) at approximately 490°C [25, 26, 27]. This symmetry‐lowering transition is driven by the cooperative Jahn‐Teller effect associated with Cu^2+^ ions. In an octahedral ligand field, Cu^2+^ (3d^9^) has an electronically degenerate ground state in the *e_g_* orbital manifold. To remove this degeneracy, each CuO_6_ octahedron undergoes an elongation distortion, a classic Jahn‐Teller effect, which in isolation would be purely local.

However, in a crystalline lattice, these local distortions can become cooperative, propagating in a long‐range, symmetry‐breaking manner that leads to a globally tetragonal phase. This cooperative distortion is stabilized when the electronic energy gain from lifting orbital degeneracy exceeds both the entropy gain of the high‐symmetry phase and the elastic penalty of lattice deformation. As a result, the cubic‐to‐tetragonal transition in CuFe_2_O_4_ is an archetypal example of a CJT‐driven structural phase transition, making it an ideal candidate to study how external stimuli, such as MW fields, can modulate or suppress such cooperative behavior.

Additionally, CuFe_2_O_4_ is structurally and chemically robust, magnetically ordered, and compatible with multiple synthesis routes, including sol–gel, hydrothermal, and microwave methods [28, 29, 30]. This makes it not only mechanistically interesting but also experimentally tractable for investigating the decoupling of local and long‐range order, a key feature of MW interaction with symmetry‐lowering phenomena.

In this study, we use CuFe_2_O_4_ as a model system to investigate how MW annealing interferes with cooperative symmetry‐lowering transitions, particularly the cubic‐to‐tetragonal phase transition driven by Jahn‐Teller‐active Cu^2+^ ions. Building on the well‐established understanding of CuFe_2_O_4_'s structural response to conventional thermal treatment, we investigate how MW energy, characterized by rapid, localized heating and non‐equilibrium electromagnetic field interactions, can selectively drive oxidation and local structural relaxation while simultaneously suppressing the long‐range cooperative distortions required for a global symmetry‐lowering phase transition.

By comparing samples processed under conventional furnace annealing, microwave annealing, and combined treatments, we disentangle the interplay between cation valence state, oxygen sublattice reordering, and crystallographic phase symmetry. Our approach integrates synchrotron X‐ray diffraction (XRD), pair distribution function (PDF) analysis, and X‐ray photoelectron spectroscopy (XPS) to independently probe long‐range crystallographic order, short‐range atomic correlations, and chemical state evolution.

The key insight emerging from this work is that MW annealing enables a non‐equilibrium decoupling: it promotes oxidation and local polyhedral ordering associated with Jahn‐Teller‐active Cu^2+^, but prevents the development of long‐range cooperative distortions required for the cubic‐to‐tetragonal phase transition. This provides a direct demonstration of how external fields can stabilize symmetry‐frustrated or metastable states by interfering with collective ordering mechanisms, a concept broadly applicable to correlated and functional oxides beyond the spinel system.

## 2 Experimental Methods

### 2.1 Sample Preparation

Commercial nanocrystalline copper ferrite (CuFe_2_O_4_) powder was obtained from Sigma–Aldrich and used as the starting material for all processing routes. The as‐received powder, referred to hereafter as “ Nanocrystal,” was dark brown in color and confirmed by XRD to adopt the expected cubic spinel phase.

To explore the influence of thermal and field‐assisted processing on the phase behavior and local structure, three additional samples were prepared from the same source material using distinct annealing protocols.

In the first case, the Sigma powder was annealed in a conventional box furnace at 700°C for 30 min in air, followed by natural cooling to room‐temperature. This sample is referred to as Furnace. Additional experiments with extended annealing durations up to 18 h yielded similar tetragonal phase formation; see Figure 5.

**FIGURE 1:**
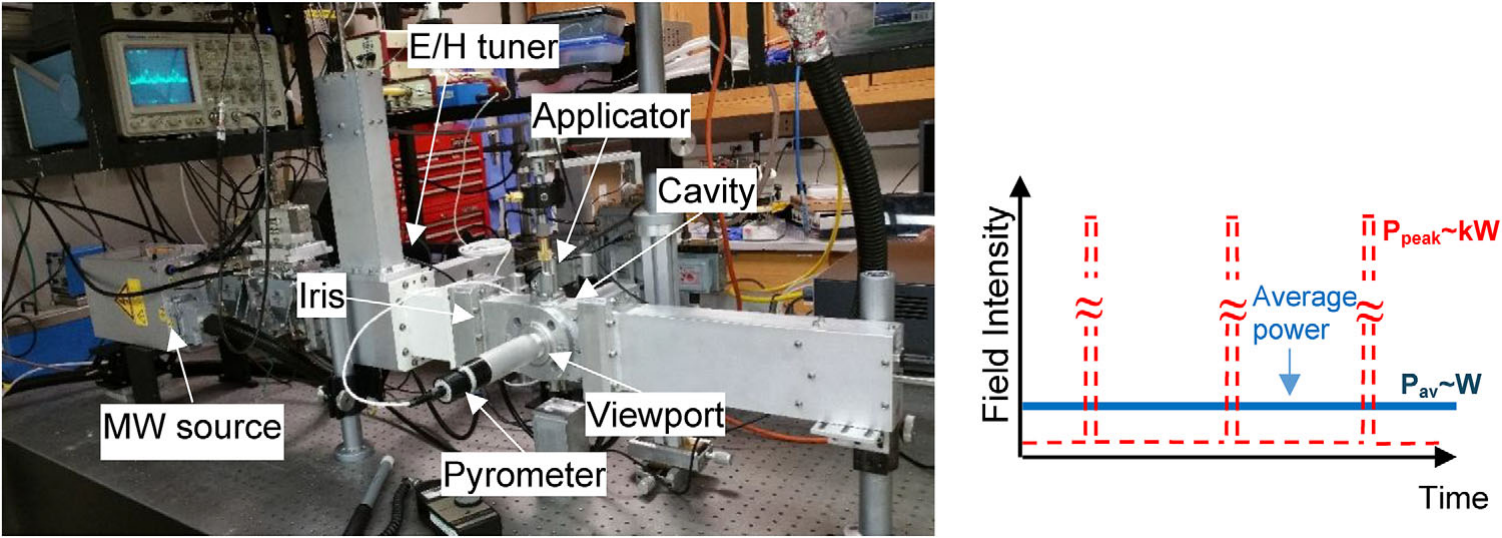
Photograph of the microwave annealing setup, including the 2.45 GHz pulsed microwave source, E/H tuner, waveguide, and single‐mode cavity where the sample is positioned. The applicator is equipped with a viewport and a pyrometer for in situ visual and temperature monitoring. The microwave source operates in pulsed mode, allowing control over the duty cycle to finely tune the average sample temperature without reducing the peak field intensity. This configuration also helps suppress thermal runaway and minimize the formation of local hot spots during processing.

In the second case, a portion of the Sigma powder was subjected to microwave annealing at 700°C, following the controlled heating and cooling profile, using a 2.45 GHz laboratory‐scale single‐mode MW cavity depicted in Figure 1. To avoid thermal or field‐induced quenching, the sample was cooled in situ by gradually reducing the MW power, allowing a controlled descent to room‐temperature under continued MW exposure. This protocol was designed not only to ensure uniform cooling but also to test whether the MW field itself suppresses the long‐range cooperative interactions required for the cubic‐to‐tetragonal phase transition. This sample is designated MW.

In the third case, the Furnace‐annealed powder was further annealed with MW at 200°C, producing a fourth sample referred to as Furnace+MW. All annealing procedures were performed without applied pressure. The process was repeated on three separate samples, and reproducibility was confirmed by XRD.

This sequence of treatments yielded four distinct samples (Sigma, Furnace, MW, and Furnace+MW), enabling direct comparison of the effects of conventional annealing, MW processing, and their combination on the structural and electronic evolution of CuFe_2_O_4_.

**FIGURE 2:**
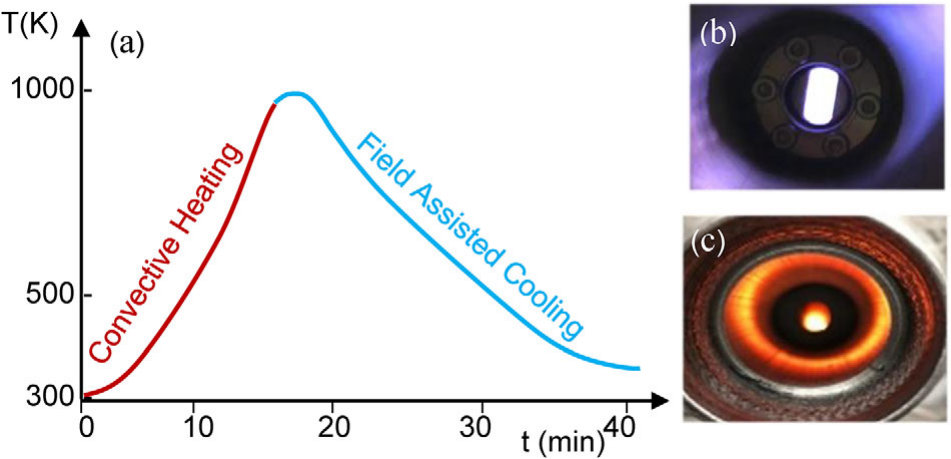
(a) Temperature profile during MW annealing. In the initial heating stage, the sample is exposed to a low‐pressure Ar plasma, where convective heat transfer from hot Ar gas, combined with surface interaction with Ar^+^ ions, raises the sample temperature to ∼700°C while minimizing direct dielectric coupling with the electromagnetic field. Once the target temperature is reached, the plasma is extinguished by increasing the Ar pressure, and dielectric heating by MW fields begins, establishing strong electric field‐material interaction. The sample is then cooled gradually under continuous MW exposure by reducing the average power, allowing sustained field interaction throughout the thermal descent. This slow, field‐assisted cooling prevents thermal quenching and promotes non‐equilibrium structural evolution. During the plasma‐heating stage, energy transfer occurs primarily via surface interactions, whereas during MW cooling, volumetric field coupling dominates. (b) Photograph of the sample through the viewport under Ar plasma heating, and (c) during field‐coupled MW cooling. The ∼20 min controlled cooling ramp excludes the possibility of kinetic quenching, which typically requires sub‐second timescales.

Furthermore, to isolate the effects of the MW electric field from purely thermal processes, a multi‐stage heating protocol was employed (Figure 2a). The CuFe_2_O_4_ powder was sealed inside an alumina crucible and placed within a quartz tube under a controlled argon atmosphere. In the initial stage, a low‐pressure Ar plasma was ignited using the MW source. The resulting hot Ar gas and Ar^+^ ions provided convective and radiative heating of the sample up to ∼700°C while simultaneously screening the sample from direct interaction with the MW electric field. This approach allowed the sample to be heated predominantly via gas‐phase mechanisms, with minimal dielectric absorption. Upon reaching ∼700°C, the gas pressure in the quartz tube was increased to atmospheric pressure to extinguish the plasma. In this high‐pressure regime, the MW field began coupling volumetrically to the sample through dielectric absorption, enabling non‐equilibrium field‐matter interactions to dominate. The sample was then cooled slowly by gradually reducing the average MW power, ensuring that the electric field remained active throughout the cooling phase. This protocol avoids both thermal and field quenching and allows the structure to evolve under sustained MW exposure during the critical cooling window. This method preserves local ordering while frustrating long‐range cooperative distortion, as explored in subsequent sections.

### 2.2 Microwave Annealing Protocol and Control of Thermal vs. Field Effects

To clarify that kinetic quenching does not contribute to the observed phase retention in our experiments, we emphasize that all microwave‐treated samples were cooled gradually from 700°C to room‐temperature over approximately 20 min (see Figure 2a). This slow, controlled thermal descent was achieved by progressively reducing the average microwave power during cooling. Such rates—many orders of magnitude slower than the extreme quenching rates (10^9^–10^10^ K s^−^^1^) typically required to kinetically trap high‐temperature metastable phases [31, 32] rule out quenching as a mechanism for phase stabilization. Moreover, EM field exposure was maintained continuously throughout the cooling period, establishing a non‐equilibrium condition distinct from both rapid quenching and conventional furnace cooling. The resulting structural states thus reflect field‐matter interactions rather than thermal rate limitations.

Fundamentally, temperature rise during MW processing is governed by the average energy delivered to the sample, whereas field‐induced (non‐thermal) effects depend on the instantaneous electric field intensity. Our custom‐built pulsed MW system^1^ exploits this distinction by delivering ultra‐short (microsecond‐scale), high‐power MW pulses at a low duty cycle. This configuration allows us to maintain high peak field strengths while minimizing the total energy deposited, significantly reducing the thermal load on the sample. In contrast to conventional continuous‐wave MW systems, where lowering power diminishes both temperature and field strength simultaneously, our setup decouples these variables, enabling independent control over heating and field exposure. The custom‐built system is capable of delivering microwave pulses with peak EM power in the megawatt range, while maintaining the average power below 100 W through low‐duty‐cycle pulsing. This configuration allows precise control over thermal input while preserving high field intensities necessary for field‐matter coupling. By preserving intense EM field‐lattice interactions under quasi‐isothermal conditions, we can selectively probe field‐driven effects on structural ordering.

Microwave annealing was carried out using a custom‐designed 2.45 GHz single‐mode cavity optimized for uniform electric field distribution and precise temperature control. The microwave power was pulsed with a user‐defined frequency and duty cycle, allowing tuning of the time‐averaged power. Pulsed operation also minimizes hot spot formation, enabling uniform heating and preventing sample damage. The specimen position inside the cavity was adjustable, allowing the sample to be placed at the electric‐field antinode where the MW field intensity is maximized. This position was identified by cavity tuning and confirmed by the thermal response of the sample, consistent with the electric‐field maximum predicted by the COMSOL simulation. Surface temperature was monitored in real time using a high‐resolution infrared (IR) camera with a magnifying lens, providing spatially resolved (micrometer‐scale) temperature mapping throughout the annealing cycle. Additional technical specifications, calibration protocols, and system validation are described in detail in our prior work [15, 16, 18].

### 2.3 Structural and Spectroscopic Characterization

Synchrotron XRD and PDF measurements were carried out at the European Synchrotron Radiation Facility (ESRF) on beamline ID15A using high‐energy X‐rays (75 keV, λ = 0.165 Å) in transmission geometry. Samples were prepared using the facility's dedicated high‐throughput sample holders, which consist of 1 mm‐thick cylindrical wells (2.5 mm in diameter) enclosed between two polyimide film windows and secured with polymer clips. Approximately 1–2 mm^3^ of finely packed powder was loaded into each well using a funnel and plunger system to ensure uniform thickness and crystallite orientation distribution. To maximize data quality and minimize preferred orientation effects, the holders were oscillated, and the beam was rastered across each well during acquisition. Sample identification was managed via QR code labeling registered to the facility's measurement workflow system.

Total scattering intensities were collected using a Pilatus CdTe 2 m area detector (1679 × 1475 pixels, 172 µm × 172 µm pixel size) positioned approximately 0.3 m downstream from the sample, with the incident beam aligned to the lower corner of the detector. Detector geometry and calibration parameters were established using a NIST SRM 660b LaB_6_ standard and refined using Rietveld analysis with known lattice parameters to determine a polynomial offset correction. Detector alignment, flat‐field response, polarization, and solid‐angle corrections were applied using *pyFAI*, and a custom pixel mask was used to exclude defective or outlier pixels.

One‐dimensional scattering profiles were obtained by azimuthal integration onto a 3000‐bin grid using a sigma‐clipping algorithm to exclude azimuthal outliers. After background subtraction, the integrated patterns were corrected for parallax and residual geometrical offsets using parameters obtained from LaB_6_ refinement in *TOPAS v7*. These corrections were propagated to all experimental datasets to ensure consistency in instrument response.

Total scattering structure functions F(Q) were generated using *PDFgetX3* with appropriate composition and sample‐dependent parameters. Fourier transforms were performed with multiple Q_max_ values (typically up to 23 Å^−1^) to assess sensitivity to high‐Q noise and termination ripples. Where noted, modification functions were applied to suppress Fourier termination effects and validate the robustness of structural features. PDF refinements in real space were performed using *PDFgui* to assess data correction quality. Fits used simple spinel models under the Morningstar–Krutter–Warren approximation [33], and residual features were interpreted in light of model simplicity. For internal consistency, LaB_6_ reference PDFs yielded typical goodness‐of‐fit values (Rw) near 9%, validating the overall quality of the measurement and reduction pipeline. Temperature‐dependent XRD measurements were performed using a PANalytical Empyrean diffractometer equipped with a high‐temperature stage to monitor phase evolution during heating and cooling. Transmission electron microscopy was performed using an aberration‐corrected FEI Titan 80–300 STEM operated in bright‐field TEM and selected area diffraction modes to assess particle size, crystallinity, and microstructural evolution.

XPS measurements were carried out at the Analytical Instrumentation Facility (AIF) at North Carolina State University using a system equipped with a PHOIBOS 150 hemispherical analyzer and an Al Kα monochromatic X‐ray source (1486.6 eV). The instrument operates with an energy resolution of < 0.1 eV and was run at a beam energy of 12 kV during acquisition. Spectra were collected under ultrahigh vacuum conditions (∼10^−9^ Torr) with a spot size of approximately 400 µm. Both survey and high‐resolution spectra were acquired, with a pass energy of 20 eV used for the Cu 2p and Fe 2p regions.

To account for charging effects, binding energies were internally calibrated to the adventitious carbon C 1s peak. The standard value for the C 1s peak from adventitious carbon is 284.8 eV. In our measurements, the C 1s peak appears at 284.9 eV for most samples, within the typical energy resolution of our XPS system (< 0.1 eV), and was corrected by ‐0.1 eV to align with the standard reference. However, the MW 700°C sample exhibited a significantly shifted C 1s peak at 285.7 eV, indicating surface charging (see the Supporting Information). Accordingly, this spectrum was corrected by ‐0.9 eV. These shifts were applied uniformly to all binding energy values reported in the Cu 2p spectra. Cu 2p spectra were analyzed to assess the oxidation state of copper via the relative intensity of shake‐up satellites associated with Cu^2+^ and the binding energy shifts associated with Cu^+^, while Fe 2p spectra were used to confirm iron valence states and monitor changes in surface composition. Data were processed using CasaXPS software.

## 3 Finite Element Modeling of the Microwave Cavity

To better understand the spatial distribution of electromagnetic energy and its thermal consequences during microwave annealing, we performed 3D finite element simulations of the single‐mode MW cavity using COMSOL Multiphysics. The goal of this modeling was to visualize the standing wave electric field pattern within the cavity, identify field maxima where the sample experiences the strongest dielectric coupling, and quantify the resulting thermal gradients along the specimen.

The model couples two physics interfaces: (1) Electromagnetic Waves, Frequency Domain and (2) Heat Transfer in Solids, allowing a self‐consistent evaluation of both field distribution and resulting temperature profiles in the specimen. The MW source is modeled at a frequency of 2.45 GHz, corresponding to our experimental setup.

### 3.1 Geometry and Boundary Conditions

The cavity geometry was constructed to reflect the experimental configuration of a WR‐340 single‐mode waveguide cavity, including a narrow iris aperture on one side and a sliding short circuit on the other. In the experimental system, the sliding short is adjusted to achieve resonance by tuning the effective cavity length to support a standing wave at 2.45 GHz. In the COMSOL model, this tunability is mimicked by adjusting the cavity length such that the simulated electromagnetic field satisfies the resonance condition for the TE_10_ mode.

In the simulation, the waveguide port with the iris was defined using a scattering boundary condition to simulate input power injection, while the sliding short boundary was treated as a perfect electric conductor (PEC) wall to reflect the incoming wave, thereby creating the standing wave condition. The remaining cavity walls were also modeled as PEC boundaries, which reflect electromagnetic energy and confine the mode structure. This setup accurately reproduces the experimental field distribution within the cavity and allows for realistic modeling of the specimen's exposure to microwave energy.

The specimen was modeled as a cylindrical sample (5 mm in diameter, 25 mm in length) composed of CuFe_2_O_4_ filled inside a quartz tube, using experimentally measured values for the real and imaginary components of the complex permittivity (εʹ and ε″) and thermal conductivity. Because the electrical conductivity and loss tangent of CuFe_2_O_4_ can increase at elevated temperature, the microwave penetration depth was considered through the complex permittivity used in the electromagnetic model. The loss component includes both dielectric polarization loss and conductive loss, so the simulated field distribution reflects attenuation within the specimen rather than assuming uniform field penetration. Although increased high‐temperature conductivity can reduce the effective skin depth, TEM shows that the MW‐treated particles are generally below or on the order of several micrometers, which is smaller than or comparable to the microwave penetration depth expected for semiconducting ferrite powders under typical 2.45 GHz processing conditions. Thus, individual particles are expected to experience field penetration rather than purely surface‐limited heating. Because the sample is a porous powder compact rather than a dense metallic conductor, interparticle contact resistance and porosity further reduce the likelihood of complete electromagnetic shielding. This supports the interpretation that full surface oxidation does not necessarily produce equivalent local bulk structural relaxation.

The tube was placed at the electric field antinode corresponding to the TE_10_ mode, where the electric field is maximized. Thermal boundary conditions included natural convection at the specimen surface and thermal insulation at the cavity walls, assuming negligible conduction to the walls due to minimal contact area.

### 3.2 Electromagnetic Field and Temperature Distribution

**FIGURE 3:**
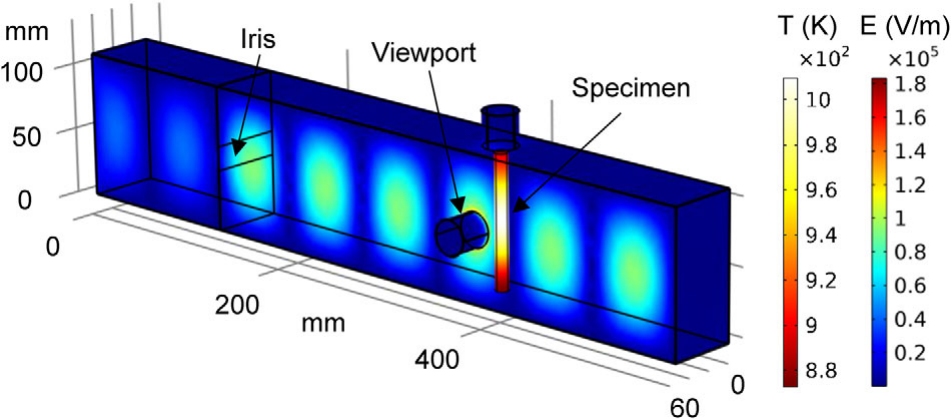
Finite element simulation of the microwave cavity using COMSOL Multiphysics, illustrating the standing wave pattern (electric field distribution) inside the single‐mode cavity and the resulting temperature profile along a rod‐shaped specimen placed at the field maximum.

Figure 3 shows the spatial electric field distribution inside the cavity, illustrating the standing wave pattern generated by the TE_10_ mode. As expected, the electric field forms a sinusoidal distribution along the cavity axis, with maxima at λ/2 intervals. The field intensity is strongly localized near the specimen center, demonstrating the volumetric energy concentration typical of single‐mode MW cavities.

The simulation confirms that the specimen experiences maximum field exposure when placed at the antinode of the standing wave. This spatially selective coupling leads to rapid internal heating, particularly in dielectric materials where the loss tangent (tan δ) is nonzero. Importantly, the field distribution is highly sensitive to specimen position, shape, and dielectric properties, highlighting the need for careful sample placement and cavity tuning in experimental work.

The coupled thermal simulation reveals that the center of the sample experiences a relatively uniform temperature, reflecting the flatter electric field distribution near the center of the standing wave. In contrast, steeper temperature gradients appear near the cavity walls, where the electric field rapidly diminishes due to proximity to the PEC boundaries. The hottest region of the sample aligns with the electric field antinode (maximum), confirming the expected correlation between field intensity and dielectric heating.

Under steady‐state MW exposure, the simulation confirms that the sample experiences relatively uniform heating along its length, particularly in the central region where the electric field distribution is flat. This field‐flat region is deliberately chosen for sample placement to minimize temperature gradients and avoid hot spot formation. Unlike conventional furnace heating, where heat flows from the surface inward, MW dielectric heating penetrates the material volumetrically, promoting internal heating and thermal uniformity. To further enhance uniformity and suppress transient hot spots, the system operates with a pulsed microwave source, allowing the sample to equilibrate during the off cycles.

These modeling results are consistent with experimental observations of uniform oxidation and local oxygen coordination rearrangement under MW treatment, even at modest external temperatures. The simulations also emphasize the importance of cavity design and field profile optimization: although the standing wave pattern naturally varies across the cavity, careful positioning of the sample at the field plateau, combined with time‐averaged heating from pulsed delivery, ensures controlled, spatially uniform thermal processing that enables selective non‐equilibrium transformations not achievable under traditional heating conditions.

These field and thermal behaviors have important implications for the structural response of the material. As we will discuss in later sections, the suppression of the cubic‐to‐tetragonal phase transition in MW‐processed CuFe_2_O_4_ is linked to the frustration of long‐range cooperative Jahn‐Teller distortions. The present simulations support this hypothesis by demonstrating how MW field delivery is spatially localized through resonance and time‐modulated in a way that promotes short‐range reordering without enabling the global lattice coherence required for symmetry‐breaking transitions. The absence of uniform strain fields and the presence of localized field‐matter coupling likely disrupt the phonon coherence and orbital‐lattice coupling necessary for cooperative distortions to propagate.

In this context, finite element modeling not only reveals how MW cavities shape energy landscapes within the sample but also highlights key processing constraints and design strategies. By optimizing cavity geometry, sample placement, and pulsing parameters, MW annealing conditions can be tailored to selectively activate local ordering pathways while frustrating cooperative transitions. As demonstrated here and explored in the following sections, this enables stabilization of metastable or symmetry‐frustrated phases that are inaccessible through conventional thermal processing.

## 4 Results and Discussion

### 4.1 Phase Identification by X‐Ray Diffraction

**FIGURE 4:**
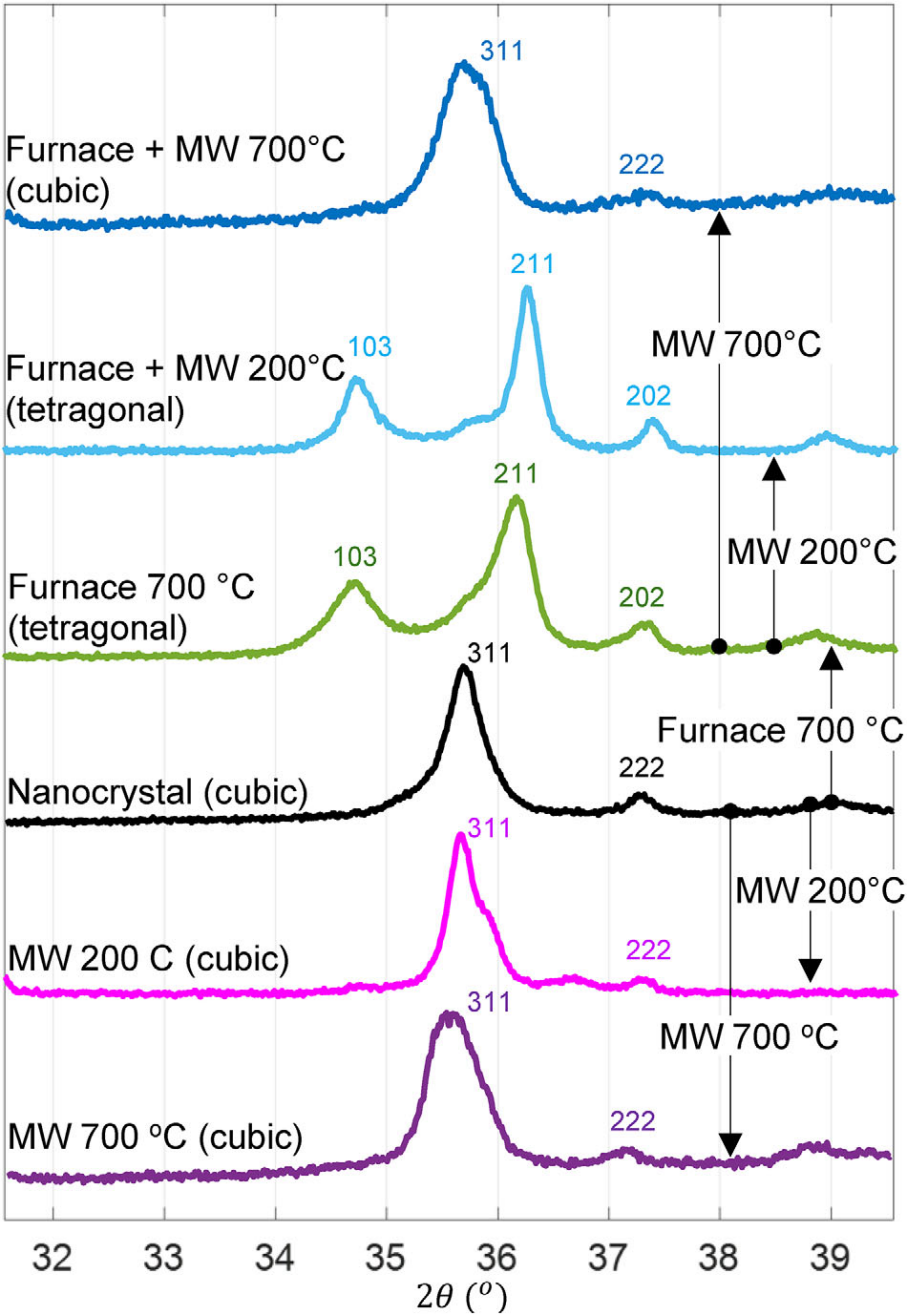
X‐ray diffraction patterns of the six CuFe_2_O_4_ samples measured at room‐temperature: Nanocrystal (as received), MW 200°C (microwave annealed at 200°C), MW 700°C (microwave annealed at 700°C), Furnace (conventionally annealed at 700°C), Furnace+MW 200°C (Furnace sample subsequently MW‐annealed at 200°C), and Furnace+MW 700°C (Furnace sample subsequently MW‐annealed at 700°C). The Nanocrystal, MW 200°C, MW 700°C, and Furnace+MW 700°C samples exhibit patterns consistent with the cubic spinel structure ($\mathrm{Fd}\bar{3}m$), while the Furnace and Furnace+MW 200°C samples show peak splitting indicative of the tetragonal phase (I4_1_/amd). Notably, despite being oxidized and annealed at the same temperature as the Furnace sample, both the MW 700°C and Furnace+MW 700°C samples retain cubic symmetry upon cooling, suggesting that MW exposure suppresses the cooperative Jahn‐Teller interactions responsible for the symmetry‐lowering phase transition. The displayed 2θ range is intentionally limited (≈32–39°) to highlight peak splitting associated with tetragonal distortion; Full‐range diffraction patterns, including the (400) and (440) reflections, are provided in the Supporting Information.

The crystallographic structures of the six CuFe_2_O_4_ samples were investigated using XRD at room‐temperature. Figure 4 shows the resulting diffraction patterns for the Nanocrystal (initial), MW 200°C, MW 700°C, Furnace, Furnace+MW 200°C, and Furnace+MW 700°C samples. Distinct differences in peak symmetry, splitting behavior, and reflection intensities reveal key differences in the structural evolution of these materials as a function of processing route.

The Nanocrystal sample, composed of commercially sourced CuFe_2_O_4_ powder, exhibits diffraction peaks characteristic of the high‐symmetry cubic spinel structure (space group $\mathrm{Fd}\bar{3}m$). The absence of peak splitting, particularly in the 311 reflection within the displayed range, indicates that the structure remains cubic despite the presence of Jahn‐Teller‐active Cu^2+^ ions. This is consistent with the nanocrystalline grain size of the sample, which frustrates the propagation of long‐range CJT distortions due to limited coherence length and dominant surface effects.

Both the MW 200°C and MW 700°C samples also show cubic spinel diffraction patterns at room‐temperature, closely resembling the Nanocrystal. This result is particularly notable for the MW 700°C sample, which was annealed at a temperature known to induce the tetragonal phase under conventional heating. The fact that no splitting is observed in the 400 or 440 reflections confirms that even high‐temperature MW annealing fails to trigger the cubic‐to‐tetragonal transition. These observations strongly suggest that the MW field suppresses the cooperative orbital‐lattice interactions necessary for global symmetry breaking, even while enabling oxidation and short‐range reordering.

In contrast, the Furnace and Furnace+MW 200°C samples exhibit clear peak splitting in the 400 and 440 reflections, consistent with a transition to the tetragonal phase (space group I4_1_/amd). This structural distortion originates from long‐range CJT ordering of Cu^2+^O_6_ octahedra and indicates that conventional furnace annealing provides sufficient thermal equilibration and atomic mobility to support cooperative distortions. Notably, the Furnace+MW 200°C sample retains the tetragonal structure, indicating that low‐temperature MW treatment following conventional annealing does not reverse the CJT phase once established.

A particularly instructive comparison arises with the Furnace+MW 700°C sample, which exhibits a return to cubic symmetry despite beginning as a tetragonal phase. The diffraction pattern of this sample lacks peak splitting and is nearly identical to the MW 700°C sample. This result demonstrates that re‐exposure to high‐temperature MW treatment not only suppresses the formation of CJT ordering but can also erase it after it has been established, effectively re‐symmetrizing the structure. This recovery of cubic symmetry under MW exposure strongly supports the hypothesis that MW fields interfere with the phonon‐mediated coupling required for CJT propagation, especially during field‐assisted cooling.

The present data do not establish a universal threshold in MW field intensity, duty cycle, or exposure time for re‐symmetrization. Rather, they show that under the specific condition of MW exposure at 700°C followed by slow field‐assisted cooling, a previously tetragonal Furnace sample can revert to cubic symmetry, whereas low‐temperature MW treatment at 200°C does not reverse the tetragonal phase. This indicates that re‐symmetrization is both temperature‐ and field‐history‐dependent. A full reversibility map would require cyclic furnace/MW treatments and systematic variation of field strength, duty cycle, pulse duration, and cooling rate, which is beyond the scope of the present study. As this work represents an initial demonstration of field‐induced re‐symmetrization in this system, more detailed parametric and mechanistic studies will be needed to fully establish the conditions governing reversibility and threshold behavior.

Taken together, these XRD results show that four samples (Nanocrystal, MW 200°C, MW 700°C, and Furnace+MW 700°C) remain cubic, while two samples (Furnace and Furnace+MW 200°C) exhibit the tetragonal phase. Crucially, these differences cannot be attributed solely to annealing temperature or oxidation state, as all samples were processed at or near 700°C. Instead, the results emphasize that the microwave field acts as a non‐equilibrium perturbation that decouples local symmetry‐breaking mechanisms from the long‐range coherence required for global structural transitions. As such, MW processing offers a pathway to stabilizing metastable cubic phases in systems that would otherwise undergo cooperative symmetry lowering under thermal equilibrium.

To further compare line broadening across samples, coherent crystallite sizes were estimated using the Scherrer equation and are summarized in Table 1. The Nanocrystal sample exhibits the smallest coherent domain size (∼17 nm), while furnace annealing increases the apparent crystallite size to ∼31 nm. MW‐treated samples show intermediate values (∼21–26 nm), indicating partial domain growth relative to the starting powder but less narrowing than expected for purely thermal coarsening. In particular, both MW 700°C and Furnace+MW 700°C exhibit similar coherent domain sizes (∼26 nm), despite their different structural histories. This suggests that high‐temperature MW processing involves competing effects of thermal coarsening and field‐induced disruption of long‐range coherence, consistent with the retention or recovery of cubic symmetry under MW exposure [16].

**TABLE 1:** Summary of structural, spectroscopic, and mechanistic features of the six CuFe_2_O_4_ samples investigated. While all samples contain Cu^2+^, their phase symmetry, local CuO_6_ environments, and XPS spectral features differ significantly based on processing history. Nanocrystal and MW‐treated samples remain cubic but for fundamentally different reasons, finite‐size frustration vs. field‐induced suppression of CJT distortion. The Furnace and Furnace+MW 200 °C samples exhibit tetragonal symmetry due to the development and retention of long‐range CJT order. In contrast, the Furnace+MW 700°C sample undergoes a phase reversal from CJT‐tetragonal to cubic under MW cooling, leading to a structurally cubic but electronically decoupled phase with reduced shake‐up intensity.

Feature	Nanocrystal (Initial)	MW 200 °C	MW 700 °C	Furnace 700 °C	Furnace+MW 200 °C	Furnace+MW 700 °C
**Structural origin**	Finite‐size frustration prevents CJT	Surface reordering without global change	Never entered CJT state	Cooperative JT distortion fully developed	CJT state retained after low‐T MW	Relaxed from tetragonal CJT state
**XRD phase**	Cubic	Cubic	Cubic	Tetragonal	Tetragonal	Cubic
**CuO_6_ environments**	Local JT‐active, distorted at surface	Similar to nanocrystal	Local JT‐active, possibly elongated	Long‐range cooperative JT‐distorted	Retains distorted CJT structure	Decoupled or symmetrized after CJT collapse
**Scherrer coherent domain size (nm)**	17	21	26	31	25	26
**Shake‐up satellite (XPS)**	Strong (enhanced by surface disorder)	Strong (similar to nanocrystal)	Strong	Strongest (max Cu^2+^ surface content)	Similar to Furnace	Reduced
**XPS main peak (2p_3/2_)**	Highest (936.8 eV, charging)	Lower (933.3 eV)	Lowest (932.3 eV)	Mid (935.0 eV)	Slightly lower (934.2 eV)	Lower (933.2 eV)
**MW‐specific O‐O/ polyhedral correlation**	Absent	—	Present	Absent	—	—
**Interpretation**	Nanostructure suppresses CJT	MW promotes local reordering but no CJT	MW preserves JT‐active Cu^2+^, blocks CJT	Conventional route to CJT phase	CJT preserved during mild MW	CJT collapse under MW suppresses coherence

**FIGURE 5:**
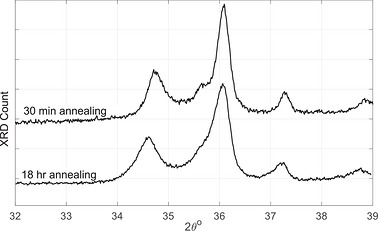
X‐ray diffraction patterns comparing the nanocrystal sample after annealing in a conventional furnace at 700°C for 30 min (top) and 18 h (bottom). Both treatments result in nearly identical diffraction features consistent with the tetragonal phase.

To further confirm that the tetragonal phase observed in conventionally annealed samples is not limited to a specific duration or thermal history, we conducted additional furnace annealing experiments on the nanocrystal sample at 700°C for both short (30 min) and extended (18 h) durations. As shown in Figure 5, both treatments result in diffraction patterns with nearly identical peak splitting and profile shapes, characteristic of the tetragonal CuFe_2_O_4_ phase. This reinforces that, under conventional thermal equilibrium, oxidation and cooperative Jahn‐Teller distortion robustly drive the cubic‐to‐tetragonal transition, regardless of annealing time. In contrast, as we discussed above, samples subjected to microwave treatment at the same temperature followed by field‐assisted cooling fail to exhibit this symmetry‐lowering transition, showing a fundamentally different mechanism of structural evolution under microwave fields.

### 4.2 Local and Intermediate‐Range Order From Pair Distribution Function (PDF) Analysis

To probe structural order beyond the average crystallographic symmetry revealed by XRD, we performed total scattering measurements and extracted real‐space PDFs for three selected samples, namely, Nanocrystal, Furnace 700°C, and MW 700 °C. These PDFs provide direct insight into local and intermediate‐range atomic correlations, including metal–oxygen, oxygen–oxygen, and metal‐metal distances, which are especially sensitive to Jahn‐Teller distortions and oxygen sublattice reordering.

**FIGURE 6:**
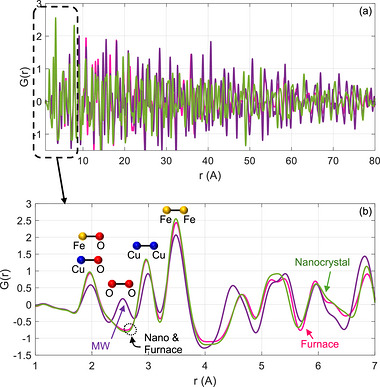
(a) Pair distribution functions of the three CuFe_2_O_4_ samples (Nanocrystals, Furnace 700°C, and MW 700°C) plotted over the range 1–80 Å, showing the extent of long‐range structural coherence. (b) Expanded view of the short‐range region (1–7 Å), highlighting the first three peaks associated with metal–oxygen and oxygen–oxygen distances. The Cu─O/Fe─O peak at ∼1.9–2.0 Å is present in all samples, indicating robust local polyhedral structure. The O–O correlation at ∼2.5 Å is clearly visible in the MW sample but absent in the Nanocrystal and Furnace samples, revealing the role of MW annealing in modifying local O–O/polyhedral correlations without inducing the global cubic‐to‐tetragonal phase transition.

The full‐range PDFs from 1 to 80 Å are shown in Figure 6a, while the zoomed‐in view of the short‐range region (1 to 7 Å) is shown in Figure 6b. The first major peak in the PDF, located near 1.9–2.0 Å across all samples, corresponds to nearest‐neighbor Cu─O and Fe─O bonds within the spinel framework. The position and sharpness of this peak indicate that short‐range cation‐oxygen polyhedra remain well‐formed regardless of annealing method, reflecting the intrinsic stability of the metal–oxygen bond lengths in CuFe_2_O_4_. No significant shifts in peak position were observed between samples, suggesting that oxidation states and octahedral/tetrahedral site geometries remain locally similar in all cases, in agreement with XPS results presented later.

A key distinguishing feature appears near ∼2.5–2.6 Å in Figure 6b and is clearly visible only in the MW‐treated sample. To support the assignment of this feature, PDFgui refinements were performed for the Nanocrystal, Furnace 700°C, and MW 700°C samples using cubic and tetragonal spinel‐based structural models (Figures S3–S6). The fits confirm that the short‐range region contains overlapping contributions from metal–oxygen, O–O, and polyhedral pair correlations, but the additional feature observed in the MW‐treated sample is most consistent with an oxygen‐sensitive O–O/polyhedral correlation arising from local oxygen displacement and coordination rearrangement. The MW sample also exhibits a larger refined atomic displacement parameter, consistent with a broader distribution of local oxygen environments [18, 34, 35]. Thus, the MW‐specific feature is interpreted as evidence of local oxygen‐polyhedral rearrangement rather than simple long‐range oxygen sublattice ordering.

Although the MW‐specific short‐range feature indicates local oxygen coordination changes, it alone does not explain the suppression of the tetragonal phase. The strongest evidence for field‐assisted disruption of cooperative Jahn‐Teller ordering is the recovery of cubic symmetry in the Furnace+MW 700°C sample, which originates from a previously tetragonal CJT‐ordered state with grain sizes well above the finite‐size limit for cooperative ordering. Thus, while MW‐enhanced oxygen mobility and local defect relaxation likely contribute to local structural rearrangement, they cannot by themselves account for the reversal of long‐range symmetry lowering observed after MW exposure.

Additional peaks in the PDF between 3 and 5 Å correspond to intermediate‐range metal–metal correlations such as Cu–Fe, Cu–Cu, and Fe–Fe distances within edge‐sharing or corner‐sharing octahedra and tetrahedra. These features are observed in all three samples studied but differ in sharpness and position, reflecting variations in intermediate‐range ordering and local distortions.

In both the Nanocrystal and Furnace samples, these peaks are relatively sharp and occur at consistent positions, indicating well‐defined polyhedral connectivity and comparable intermediate‐range order. Despite their differing global symmetries (cubic vs. tetragonal), these two samples appear to retain a similar degree of short‐ and medium‐range metal–metal ordering, possibly due to the high structural rigidity of the spinel framework and the dominance of corner‐sharing geometries that remain relatively unperturbed by the cooperative Jahn‐Teller distortion.

In contrast, the MW 700°C sample exhibits a noticeable shift in the peak near 4.5 Å along with increased peak broadening. This broadening suggests a distribution of bond lengths and a higher degree of local structural disorder, consistent with local relaxation and oxygen‐related coordination rearrangement under MW treatment. The shift may also indicate subtle rearrangements in octahedral and tetrahedral connectivity, reflecting MW‐induced modification of local oxygen coordination environments or strain accumulation from suppressed cooperative distortions.

These findings underscore the unique ability of MW processing to promote local reorganization while inhibiting the development of coherent long‐range symmetry breaking.

At longer real‐space distances (r > 20 Å), all samples exhibit sustained PDF oscillations extending to ∼80–100 Å, confirming the presence of long‐range structural coherence and ruling out any amorphous or highly disordered phases. However, the rate of damping varies among the samples. The MW PDF shows slightly slower attenuation of oscillation amplitude, indicating sustained intermediate‐range coherence despite increased local coordination disorder.

Taken together, the PDF analysis shows that microwave annealing modifies the local oxygen‐polyhedral environment and enhances a distinct short‐range O–O‐related correlation without inducing the long‐range cooperative distortion required for tetragonal symmetry. In contrast, conventional furnace annealing produces the expected tetragonal CJT phase but does not exhibit the same MW‐specific local oxygen correlation. This distinction reinforces that local oxygen‐polyhedral rearrangement and long‐range cooperative symmetry breaking are related but separable structural responses.

### 4.3 Cu and Fe Oxidation States From XPS

**FIGURE 7:**
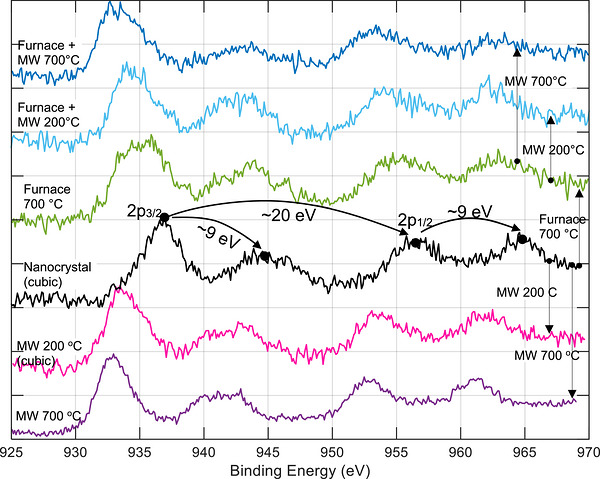
Cu 2p X‐ray photoelectron spectra for six CuFe_2_O_4_ samples: Nanocrystal (initial), MW 200°C, MW 700°C, Furnace (annealed at 700°C), Furnace+MW 200°C, and Furnace+MW 700°C. All spectra exhibit characteristic Cu^2+^ features, including a 2p_3/2_ main peak near 933–934 eV and a shake‐up satellite approximately 9 eV higher in binding energy. The Nanocrystal sample shows a uniform positive energy shift of ∼3 eV, attributed to surface charging due to its high surface area and poor conductivity. The Furnace sample exhibits the highest shake‐up satellite, consistent with high Cu^2+^ content following conventional oxidation at 700°C. Notably, the MW 700°C and Furnace+MW 700°C samples, despite both being cubic by XRD and similarly broadened, display markedly different satellite intensities. This contrast is attributed to differences in their structural histories: the MW 700°C sample retains locally JT‐active Cu^2+^ due to the absence of prior cooperative ordering, while the Furnace+MW 700°C sample undergoes field‐induced suppression of orbital coherence during cooling, reducing satellite intensity.

To probe the oxidation states and local electronic environment of copper and iron in the CuFe_2_O_4_ samples, we performed XPS, focusing primarily on the Cu 2p core level. Representative Cu 2p_3/2_ spectra for the six processed samples are shown in Figure 7. All samples exhibit characteristic Cu^2+^ features: a main 2p_3/2_ peak near 933–934 eV accompanied by a shake‐up satellite ∼9 eV higher in binding energy, consistent with the 3d^9^ final state of Cu^2+^.

However, the exact binding energies and satellite intensities vary systematically with thermal history and processing conditions, providing insights into surface oxidation, charging effects, and structural evolution. The Nanocrystal (initial) sample shows the 2p_3/2_ peak centered at 936.8 eV, ∼3 eV higher than in other samples. This uniform shift across all features is attributed to surface charging, a common effect in fine‐grained or poorly conducting powders with large surface areas. Supporting this interpretation, the C 1s peak also appears shifted to ∼286 eV in this sample, relative to the expected 284.8 eV in the others (not shown in the figure).

While the MW 200°C and MW 700°C samples show reduced binding energies compared to the Nanocrystal sample, indicative of decreased surface charging, the full widths of the main Cu 2p_3/2_ peaks remain essentially unchanged. This suggests that MW treatment does not significantly reduce local chemical or structural disorder around Cu at the surface. Instead, the observed shift is best attributed to modest grain growth and reduced surface adsorbates, which improve surface conductivity and charge dissipation during XPS measurement, rather than to any meaningful narrowing of the Cu coordination environment.

The Furnace 700°C sample exhibits the highest satellite peak relative to the main 2p_3/2_ peak, indicating the highest concentration of surface Cu^2+^ among all samples. This is expected given the sustained high‐temperature annealing in air, which promotes complete surface oxidation. Notably, the main Cu 2p_3/2_ peak in this sample is also slightly broader than in other samples. This broadening could reflect either a distribution of chemical environments caused by oxygen inhomogeneity or residual multiplet splitting due to unrelaxed local distortions, consistent with partial bulk disorder revealed in the PDF analysis.

The Furnace+MW 200°C sample closely resembles the Furnace sample in both peak position and satellite structure, suggesting that post‐furnace MW treatment at low temperature does not significantly alter the surface oxidation state. However, the Furnace+MW 700°C sample shows a noticeable reduction in satellite intensity, with its Cu 2p_3/2_ peak shifted to 933.2 eV, among the lowest binding energies observed. This observation is striking when contrasted with the MW 700°C sample, which shows a similarly low binding energy but retains strong satellite features.

Although both the MW 700°C and Furnace+MW 700°C samples are cubic by XRD and exhibit comparable crystallite sizes based on FWHM broadening, their Cu 2p satellite intensities differ significantly. We attribute this discrepancy to differences in their thermal and structural histories. The MW 700°C sample is directly annealed from the Nanocrystal precursor and never passes through a CJT phase. As a result, it retains locally JT‐active Cu^2+^ centers, which support strong ligand‐to‐metal charge‐transfer transitions and thus pronounced satellite peaks.

In contrast, the Furnace+MW 700°C sample begins in the tetragonal CJT‐ordered phase and reverts to a cubic phase only after MW treatment. This sequence of processing disrupts orbital coherence and likely relaxes or symmetrizes the CuO_6_ octahedra, reducing the efficiency of shake‐up transitions and suppressing the satellite intensity. These findings suggest that MW exposure, when applied after CJT ordering has already occurred, can erase cooperative distortions and leave behind a structurally cubic yet electronically decoupled phase. This subtle but revealing contrast between the two cubic samples, one field‐prevented and the other field‐erased, warrants deeper analysis. We explore this mechanistic distinction further in the next section.

Taken together, these results show that all samples exhibit surface features consistent with Cu^2^^+^, but that the intensity and shape of the satellite structure are highly sensitive to processing route, thermal history, and surface coordination. The Nanocrystal sample, for instance, shows evidence of charging and possibly mixed‐valence Cu^+^/Cu^2+^, while the MW and Furnace samples reflect varied degrees of oxidation and electronic disorder. Crucially, these surface‐sensitive XPS results must be interpreted alongside the bulk‐sensitive structural data presented in Sections 4.1 and 4.2.

For example, the Furnace sample, despite showing the most intense surface Cu^2+^ signal, lacks the O–O peak at 2.5 Å in its PDF, indicating that local oxygen polyhedral order is not fully restored in the bulk. This supports the interpretation that full surface oxidation does not guarantee bulk reordering. Grain growth during furnace annealing likely reduces oxygen diffusivity, leaving the interior in a kinetically trapped disordered state. The broader 2p_3/2_ peak in this sample may reflect such underlying structural inhomogeneity.

In contrast, the MW 700°C sample, though lacking tetragonal distortion in XRD, shows strong O–O correlations in PDF, implying substantial modification of local bulk oxygen coordination. This aligns with the known ability of MW irradiation to enhance oxygen mobility and defect healing via non‐equilibrium energy delivery. Nevertheless, the sample remains globally cubic, revealing a core finding of this work: MW fields can enable oxidation and local symmetry breaking without triggering the long‐range orbital‐lattice coherence needed for cooperative Jahn‐Teller distortion.

In addition to the Cu 2p spectra, the Fe 2p XPS data further corroborate the influence of microwave processing on the local electronic environment (see Figure S1). While all samples exhibit Fe^3+^ features with consistent spectral shape, indicating no measurable presence of Fe^2+^, small but systematic shifts in binding energy are observed across the series. The Nanocrystal sample displays the highest Fe 2p binding energy, whereas the MW 700°C sample shows the lowest, followed closely by MW 200°C. These shifts mirror the trends seen in the Cu 2p core level and reveal subtle differences in the electrostatic potential at the Fe site. In the Nanocrystal sample, the elevated binding energy likely reflects surface charging due to its high surface area, abundant adsorbates, and limited electronic conductivity. Additionally, undercoordinated and disordered surface Fe sites may reduce final‐state screening efficiency, further increasing the apparent core‐level energy. Conversely, the lower binding energy observed in the MW samples suggests that MW annealing promotes surface relaxation, reduces defect density, and enhances local electronic screening. These effects may arise from field‐enhanced diffusion and grain connectivity, leading to more uniformly coordinated and electronically stable Fe environments at the surface. Notably, the similarity of this trend to that observed for Cu 2p emphasizes that MW processing systematically alters the local dielectric and electronic structure of both cation sublattices. This supports our broader conclusion that microwave annealing not only decouples local and global structural order but also modifies the final‐state electronic response, providing a distinct spectroscopic fingerprint of MW‐induced structural and electronic relaxation.

### 4.4 XPS Evidence for Path‐Dependent Electronic Decoupling in Cubic CuFe_2_O_4_

A particularly instructive aspect of this study lies in the XPS analysis of the Cu 2p core‐level spectra, which reveals a marked suppression of the Cu^2+^ satellite peak in the Furnace+MW 700°C sample compared to the Nanocrystal sample. This is unexpected, given that both samples exhibit cubic symmetry by XRD, contain Cu^2+^ as the dominant oxidation state, and show comparable crystallite sizes based on line broadening. The puzzling divergence in satellite intensity raises an important mechanistic question: how can two Cu^2+^‐rich, structurally cubic samples display such distinct spectroscopic signatures?

The resolution lies in understanding that the cubic symmetry in each sample arises from fundamentally different mechanisms. The Nanocrystal sample remains cubic because of finite‐size effects: the grains are too small to support the long‐range coherence required for CJT distortion. In such nanocrystalline systems, local Cu^2+^O_6_ octahedra may still distort individually, but these distortions are spatially uncorrelated and truncated at grain boundaries. Thus, while global symmetry breaking is suppressed, local Jahn‐Teller distortions can persist in a disordered fashion.

By contrast, the Furnace+MW 700°C sample begins in a bulk tetragonal CJT‐ordered state following conventional furnace annealing and then reverts to a cubic phase after high‐temperature MW exposure. The transition to the cubic phase is not driven by grain‐size limitations but rather by field‐induced frustration of long‐range orbital‐lattice coherence. During MW cooling, the cooperative aspect of the distortion is disrupted, while oxidation and local ordering are retained. As a result, the final cubic state emerges not from nanoscale limitations but from the active suppression of CJT distortion under non‐equilibrium field conditions.

These differences in structural evolution have direct implications for the surface environments probed by XPS. In the Nanocrystal sample, the surface is chemically active, structurally disordered, and rich in hydroxyl groups and other adsorbates. Such conditions enhance ligand‐metal hybridization and increase the probability of charge‐transfer transitions that give rise to shake‐up satellites. Furthermore, local Cu^2+^O_6_ octahedra near the surface are likely more distorted due to surface strain and undercoordination, again favoring satellite intensity.

In contrast, the surface of the Furnace+MW 700°C sample has likely undergone significant reorganization due to high‐temperature MW treatment. MW annealing is known to promote surface diffusion and recrystallization, which can lead to smoother, more ordered surfaces with reduced densities of high‐energy Cu^2+^ sites. Additionally, the MW field may relax Cu‐ligand bonding or reduce ligand‐hole formation at the surface, thereby suppressing shake‐up transitions. Even though the oxidation state remains Cu^2+^, the spectral satellite intensity diminishes due to less favorable conditions for charge‐transfer.

Beyond structural coordination, the electronic coupling between Cu and its surrounding lattice plays a critical role. In the Nanocrystal sample, uncorrelated Jahn‐Teller distortions may still allow strong orbital overlap between Cu 3d states and ligand p orbitals, facilitating robust shake‐up satellite features. In the Furnace+MW 700°C sample, however, MW‐induced suppression of orbital‐lattice coherence may relax the Cu^2+^O_6_ octahedra toward more symmetric configurations or decouple them electronically. This would reduce Cu‐ligand orbital hybridization and attenuate satellite intensity, despite similar oxidation states and crystallinity.

Taken together, these results demonstrate that not all cubic phases are electronically equivalent. The weaker Cu^2+^ satellite intensity observed in the Furnace+MW 700°C sample reflects a more electronically and structurally “quiet” surface environment, achieved through MW‐induced suppression of both structural and electronic coherence. In contrast, the Nanocrystal sample, though also cubic, remains spectroscopically active due to surface disorder and localized orbital distortions. This divergence provides a rare spectroscopic insight into how MW fields selectively erase long‐range cooperative ordering while preserving local symmetry breaking, a core mechanism by which MW processing stabilizes metastable or symmetry‐frustrated states in correlated oxides.

Another notable observation is the significantly lower binding energy for the MW 700°C sample. After correcting for surface charging based on the C 1s adventitious carbon peak (see Supporting Information), the Cu 2p_3/2_ position in the MW 700°C sample appears at 932.4 eV, substantially lower than in all other samples, including the Nanocrystal and Furnace+MW 700°C cases. This raises a compelling question: if all samples contain Cu^2+^, as confirmed by the presence of shake‐up satellites, why does the MW 700°C sample exhibit the lowest core‐level binding energy?

This anomalously low binding energy cannot be attributed to the reduction of Cu^2+^ to Cu^+^ or Cu^0^. The presence of strong shake‐up satellites, centered roughly 9 eV above the main line, is characteristic of a 3d^9^ initial‐state configuration and definitively rules out a closed‐shell Cu^+^ (3d^10^) or metallic Cu (3d^10^4s^1^) state. Nor is the shift a result of sample charging or instrumental calibration error, as all spectra have been aligned using the C 1s reference, and the instrument employed has a confirmed energy resolution better than 0.1 eV. The observed downshift must therefore reflect a real change in the electronic environment of the Cu site, one that alters either the initial‐state electronic density or, more likely, the final‐state screening of the core hole.

We propose that the unusually low binding energy in the MW 700°C sample arises from enhanced final‐state screening associated with the relaxation of Cu^2+^O_6_ octahedra during field‐assisted cooling. Unlike the Furnace+MW 700°C sample, which undergoes a CJT distortion during furnace annealing before being driven back to a cubic structure, the MW 700°C sample never enters the CJT‐ordered tetragonal state. It is annealed directly from the disordered nanocrystalline state and remains cubic throughout the heating and cooling process. This means that Cu^2+^ centers in the MW sample experience local Jahn‐Teller distortions that are electronically and structurally decoupled from one another, a condition of long‐range orbital incoherence. This electronic decoupling likely produces a more symmetric or dynamically averaged ligand field, which enhances dielectric screening of the Cu core hole and reduces the observed binding energy.

Additionally, the MW process itself may contribute to the electronic relaxation of the Cu site. The volumetric and localized heating inherent to MW annealing promotes rapid oxygen diffusion, surface reconstruction, and defect annihilation. In the case of the MW 700°C sample (originating from the high‐defect Nanocrystal state), these processes may result in a smoother, more stoichiometric surface. Undercoordinated Cu^2+^ sites, hydroxyl groups, and other high‐energy surface features typically associated with elevated binding energies are likely to be annealed out. Their relaxation contributes further to the low Cu 2p_3/2_ energy observed in this sample.

At this point, however, an apparent paradox arises. If surface reconstruction under MW exposure is responsible for the lower Cu binding energy, why does the Furnace+MW 700°C sample not exhibit the same downshift, given that it too undergoes high‐temperature MW annealing? Both samples are cubic by XRD, and both have comparable peak widths, indicating similar crystallinity. Yet, the Cu 2p_3/2_ peak in the Furnace+MW 700°C sample is centered around 933.2 eV, nearly 1 eV higher than in the MW‐only sample. How can this difference be reconciled?

The answer lies in the structural history of the two samples. While the MW 700°C sample develops its cubic structure directly from the disordered, initial nanocrystalline powder, the Furnace+MW 700°C sample begins in the tetragonal CJT‐ordered state and transitions to a cubic phase only during MW exposure. In other words, MW 700°C “builds” the cubic phase, whereas Furnace+MW 700°C “erases” the tetragonal phase. This distinction has profound consequences for both lattice relaxation and electronic hybridization at the Cu site.

In the MW 700°C sample, the field‐assisted cooling pathway allows orbital disorder and dynamic local symmetry to emerge in the absence of prior cooperative distortions. The Cu^2+^O_6_ octahedra in this case may be more uniformly relaxed or electronically decoupled, leading to strong final‐state screening and low binding energy. In contrast, the Furnace+MW 700°C sample undergoes a collapse of previously coherent CJT ordering, potentially leaving behind residual strain, frozen‐in orbital configurations, or electronically frustrated Cu‐ligand interactions. These effects may hinder full surface relaxation and maintain higher binding energy states at the Cu site, despite the apparent structural similarity in XRD.

Further, the orbital‐lattice coupling itself may be qualitatively different. In the MW 700°C sample, the absence of coherent JT alignment may suppress 3d‐2p orbital overlap at the Cu site, lowering the electrostatic repulsion experienced by core electrons. In contrast, the Furnace+MW 700°C sample may retain some memory of this coupling, particularly if the MW transition pathway is incomplete or kinetically limited. This difference in orbital hybridization could further explain the disparity in core‐level binding energy.

Although reduced Cu 2p satellite intensity could, in principle, arise from several factors, including oxygen vacancy formation, local symmetry changes, or modified Cu–O hybridization, the combined XPS, PDF, and XRD results make an oxygen‐vacancy‐dominated mechanism unlikely. The persistence of Cu^2+^ satellite features, together with evidence of local oxygen‐related coordination rearrangement in the MW‐treated sample, instead supports a change in Cu‐ligand hybridization associated with suppression of cooperative Jahn‐Teller ordering. A more quantitative separation of charge‐transfer energy, ligand hybridization, and local symmetry effects could be achieved in future work using configuration‐interaction cluster model calculations of the Cu 2p spectra.

Taken together, these findings lead to an important conclusion: not all cubic phases are electronically or structurally equivalent. Even when crystallographic symmetry and grain size are matched, the path by which a material arrives at its final state (whether by growth from disorder or collapse from order) can leave distinct fingerprints in the local electronic structure. The MW 700°C sample, despite being cubic and oxidized like the Furnace+MW 700°C sample, presents a uniquely relaxed and electronically decoupled Cu environment, a direct consequence of MW‐induced suppression of cooperative orbital‐lattice interactions.

This realization underscores the broader significance of non‐equilibrium processing. By disrupting long‐range coherence while enabling local order, MW fields offer a pathway to stabilize phases that are inaccessible through equilibrium means. These phases are not just metastable structurally but also electronically distinct, a feature that opens new opportunities for tuning material properties through dynamic control of symmetry, hybridization, and defect chemistry.

### 4.5 Microstructural Insights from TEM

**FIGURE 8:**
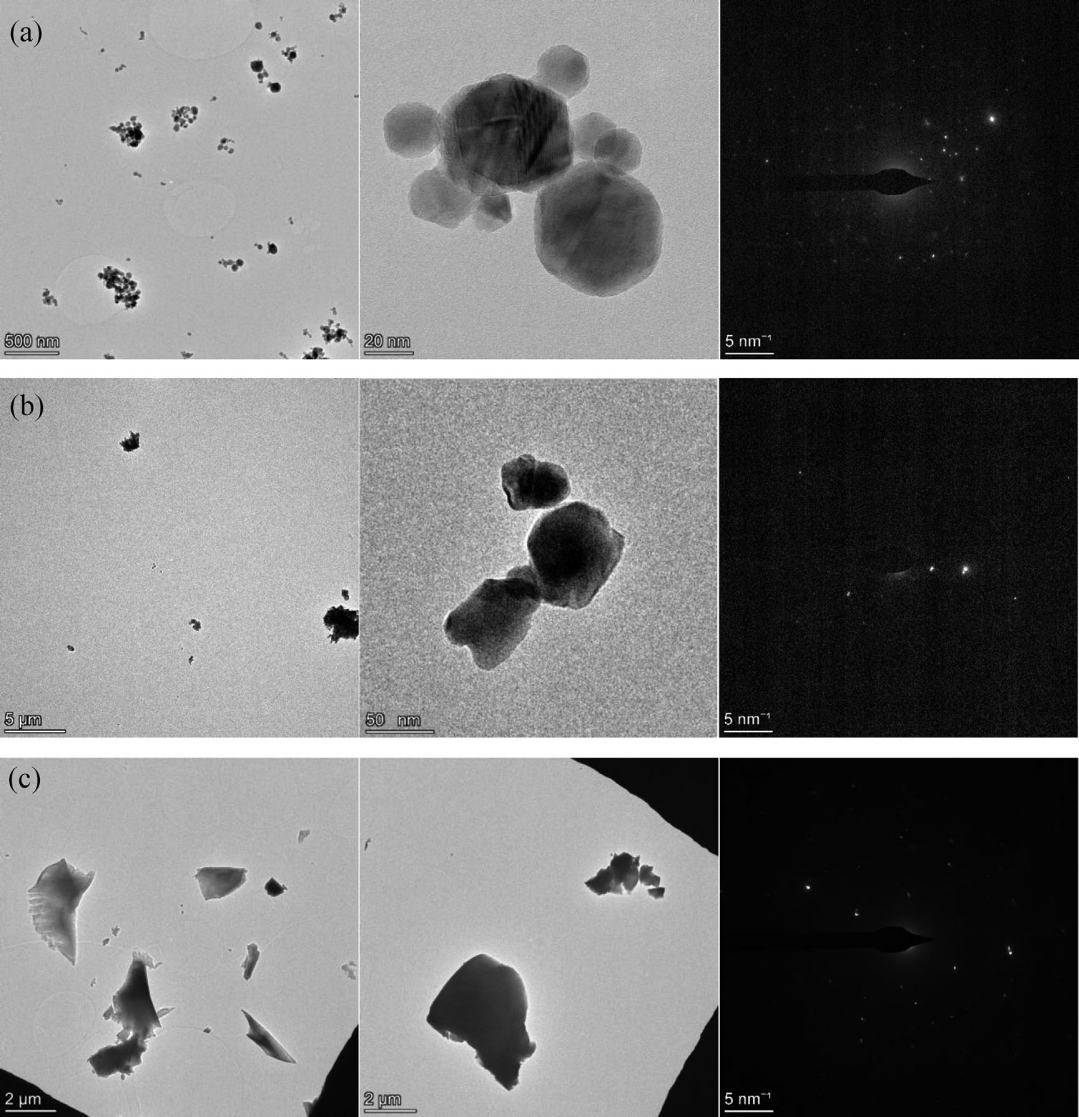
Bright‐field TEM images and corresponding selected area electron diffraction (SAD) patterns of CuFe_2_O_4_ samples: (a) Nanocrystal (as received), (b) Furnace‐annealed nanocrystal (700°C, 30 min), and (c) furnace‐annealed followed by microwave‐annealed nanocrystal (700°C each). The Nanocrystal sample exhibits particle sizes below 50 nm, indicative of a fine‐grained nanostructure. Furnace annealing increases the particle size to above 50 nm, while the Furnace+MW sample shows significant grain growth, with particle sizes ranging from several hundred nanometers to a few microns. All samples display well‐defined diffraction spots in their SAD patterns, consistent with single‐crystal or nearly single‐crystal domains.

As shown in Figure 8, transmission electron microscopy (TEM) imaging, provides complementary microstructural evidence for the phase behavior observed by XRD. We note that TEM is used here primarily to evaluate particle size, crystallinity, and microstructural evolution; evidence for local oxygen coordination changes is instead provided by the PDF analysis in Section 4.2, particularly through the MW‐specific short‐range feature near ∼2.5–2.6 Å. The Nanocrystal sample, as received, exhibits grain sizes below 50 nm, consistent with a nanocrystalline morphology that inhibits the development of long‐range orbital‐lattice coherence. This finite‐size effect explains the persistence of the cubic phase in this sample despite the presence of Jahn‐Teller‐active Cu^2+^ ions. In contrast, both the Furnace and Furnace+MW samples display significantly larger grain sizes, with the Furnace sample exceeding 50 nm and the Furnace+MW sample reaching up to several microns. The increased crystallite size in these samples would ordinarily enable the propagation of cooperative Jahn‐Teller distortions required for a cubic‐to‐tetragonal phase transition. Indeed, the Furnace sample shows the expected symmetry lowering to the tetragonal phase. However, the Furnace+MW sample, despite its large grains, remains cubic. This result confirms that the retention of cubic symmetry in the Furnace+MW sample cannot be attributed to size effects and must instead originate from microwave‐induced suppression of long‐range orbital‐lattice ordering. The TEM data thus reinforce the central conclusion that MW fields can override the intrinsic symmetry‐breaking tendencies of the material by selectively frustrating cooperative distortion mechanisms, even in microcrystalline regimes where such transitions would normally proceed.

### 4.6 Suppression of Cooperative Jahn‐Teller Distortion by MW Annealing

The preceding sections have shown that while both conventional furnace and MW annealing lead to Cu^2+^ oxidation and local structural reordering, only the furnace‐treated samples undergo a full cubic‐to‐tetragonal phase transition. The MW‐treated sample remains cubic even when processed at 700°C and cooled slowly under continuous MW exposure, a temperature and oxidation condition that, under conventional thermal treatment, reliably produce the tetragonal phase. This result reveals a central and surprising conclusion: MW annealing suppresses the development of long‐range CJT distortion, despite enabling the local conditions required for it.

The cubic‐to‐tetragonal phase transition in CuFe_2_O_4_ is driven by cooperative distortions of Jahn‐Teller‐active Cu^2+^O_6_ octahedra, which lower the crystal symmetry through orbital‐lattice coupling. As shown in Section 4.1, the Furnace and Furnace+MW samples exhibit peak splitting in XRD, indicating that these cooperative interactions have propagated throughout the lattice to stabilize a long‐range tetragonal structure. In contrast, the MW sample shows no such splitting, remaining globally cubic even though the Cu 2p XPS spectra confirm full oxidation to Cu^2+^ and the PDF analysis reveals short‐range oxygen‐polyhedral ordering, including the reemergence of the O–O correlation at 2.5 Å.

**FIGURE 9:**
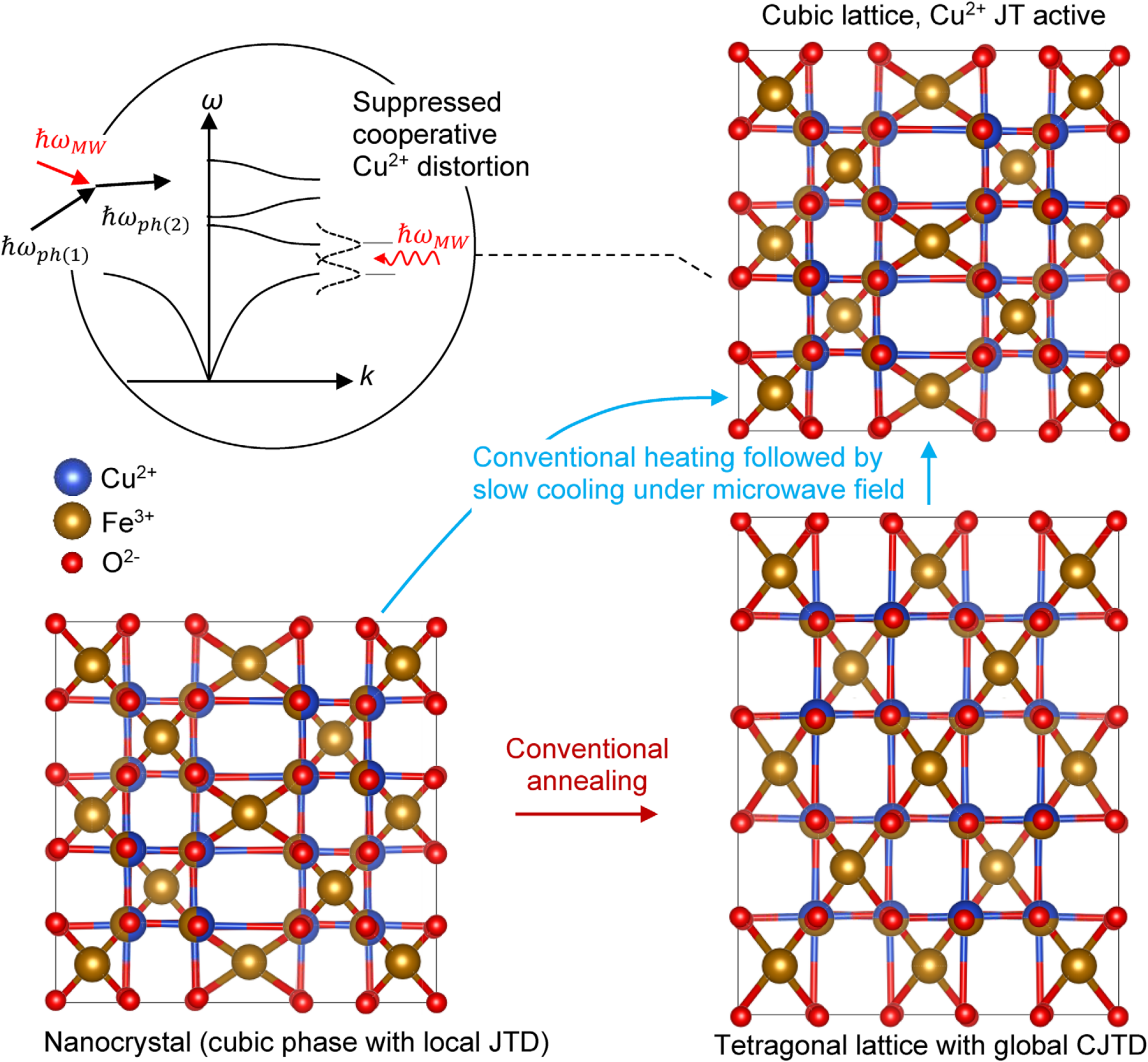
Schematic illustration of microwave‐induced suppression of cooperative Jahn‐Teller distortion in CuFe_2_O_4_. Under conventional furnace annealing, Jahn‐Teller‐active Cu^2+^O_6_ octahedra undergo local elongations that propagate coherently through the lattice, driving a global cubic‐to‐tetragonal phase transition via cooperative Jahn‐Teller distortion. In contrast, MW annealing enables oxidation and preserves local Jahn‐Teller distortions, but suppresses the long‐range coherence needed for CJT. This occurs via inelastic coupling between MW photons and thermally broadened phonon modes: MW fields may modify phonon populations through nonlinear or anharmonic interactions and may selectively depopulate lattice vibrations involved in coordinating distortions across the crystal. As a result, CJT is frustrated, and the structure is kinetically frozen into a metastable cubic phase that retains local distortions but lacks global symmetry breaking. Depicted here is the conceptual decoupling between short‐range orbital‐lattice ordering and long‐range cooperative distortion under MW‐phonon interactions during heating and field‐assisted cooling. The elongation of octahedra in the tetragonal phase and the local distortions in the cubic phase are exaggerated pictorially for clarity.

This behavior cannot be attributed to incomplete oxidation or lack of local symmetry breaking. Rather, it suggests that the MW field disrupts the long‐range coupling necessary for CJT distortion, despite enabling oxidation and local relaxation. We propose that this may arise from interaction between MW fields and phonon modes involved in coordinating lattice distortions. This mechanistic hypothesis is illustrated conceptually in Figure 9, which contrasts the pathways of cooperative distortion under equilibrium and non‐equilibrium (MW‐driven) conditions.

In microwave materials processing, temperature rise is governed by the average energy absorbed by the sample, while non‐thermal or field‐induced effects depend on the instantaneous EM field intensity. This distinction is typically obscured in continuous‐wave systems, where lowering the power reduces both temperature and field strength simultaneously. In contrast, our pulsed microwave system enables the delivery of ultra‐short (down to microsecond scale), high‐power pulses at a low duty cycle, maintaining high field intensities while minimizing average energy input. By delivering megawatt‐scale peak power EM pulses with less than 100 W of average energy, the system decouples thermal and field effects, enabling strong electromagnetic interactions without significant heating. This selective energy delivery is critical for exploring non‐thermal pathways, such as the suppression of cooperative lattice distortions. As a result, the sample remains near‐isothermal between pulses but continues to experience strong field‐matter coupling during each pulse. This configuration creates a distinct processing regime in which lattice distortions evolve under high‐field exposure without excessive heating. This is crucial to the observed suppression of cooperative Jahn‐Teller distortion, as the strong but transient field interactions may modify or disrupt distortion‐carrying phonon populations during the cooling phase, interfering with the development of long‐range orbital‐lattice coherence. Such field‐specific effects would not be accessible in conventional thermal protocols, further supporting our interpretation that the metastable cubic phase arises from a field‐driven, non‐equilibrium mechanism.

Microwave photons can interact with thermally populated phonons through two‐phonon difference processes, a type of inelastic scattering in which the energy of the incoming MW photon (ℏω_*MW*_) matches the energy difference between two phonon modes:

(1) $$\hbar \omega_{\mathrm{MW}}=h\omega_{\mathrm{ph}\left(2\right)}-\hbar \omega_{\mathrm{ph}\left(1\right)}$$

Here, ℏ is the reduced Planck constant, and ω_*ph*(1)_ and ω_*ph*(2)_ are the angular frequencies of two phonon branches. Near structural phase transitions, the thermal broadening of phonon populations increases the effective overlap in phonon density of states, making these difference processes more likely. The result is a selective depopulation of low‐energy phonon modes that mediate collective distortions, preventing their coherent alignment across the crystal.

One possible framework for describing such field‐modified phonon interactions is through the phonon Hamiltonian, which includes anharmonic terms:

(2) $${\hat{H}}_{\mathrm{ph}}=\sum_{q,\upsilon }\hbar \omega_{q,\upsilon }{\hat{b}}_{q,\upsilon }^{†}{\hat{b}}_{q,\upsilon }+{\hat{H}}^{\left(3\right)}+{\hat{H}}^{\left(4\right)}$$

In this expression, q is the phonon wavevector, υ indexes the vibrational branch, $\omega_{q,\upsilon }$ is the angular frequency of a phonon mode (q, ν), ${\hat{b}}_{q,\upsilon }^{†}$ and ${\hat{b}}_{q,\upsilon }$ are phonon creation and annihilation operators, respectively, ${\hat{H}}^{\left(3\right)}$ and ${\hat{H}}^{\left(4\right)}$ represent third‐ and fourth‐order anharmonic interaction terms.

The third‐order term, responsible for three‐phonon processes, takes the form:

(3) $${\hat{H}}^{\left(3\right)}=\sum V_{q1\upsilon 1,q2\upsilon 2,q3\upsilon 3}^{\left(3\right)}{\hat{b}}_{q_{1}\upsilon_{1}}{\hat{b}}_{q_{2}\upsilon_{2}}{\hat{b}}_{q_{3}\upsilon_{3}}^{†}+h.c.$$

Here, *V* ^(3)^ is the matrix element describing the interaction strength between three phonon modes, and *h*.*c*. denotes the Hermitian conjugate. These anharmonic interactions contribute to the self‐energy of phonons, and in particular to their imaginary part, which governs damping. The phonon linewidth (or damping rate) for mode ν can be expressed as:

(4) $$\Gamma_{\upsilon }\left(\omega \right)=\sum_{j_{1},j_{2}}{\left|V^{\left(3\right)}\right|}^{2}\left[n\left(\omega_{1}\right)-n\left(\omega_{2}\right)\right]\delta \left(\omega -\omega_{1}+\omega_{2}\right)$$

In this expression, $\Gamma_{\upsilon }\left(\omega \right)$ is the phonon damping rate (linewidth), *n*(ω) is the Bose‐Einstein distribution function (phonon occupation number), *j*_1_,*j*_2_ index intermediate phonon states, δ(ω − ω_1_ + ω_2_) is a Dirac delta function enforcing energy conservation during phonon scattering.

When MW fields drive such phonon–phonon interactions, they effectively increase $\Gamma_{\upsilon }\left(\omega \right)$, reducing phonon lifetimes and disrupting the correlated motion of CuO_6_ octahedra required for CJT coherence. Importantly, in our experimental protocol, the sample is cooled slowly under continued MW exposure, so this field‐modified phonon interaction may persist through the cooling phase. Eventually, at lower temperatures, the reduced atomic mobility kinetically freezes the structure into a metastable cubic phase, one in which local Jahn‐Teller activity is preserved, but long‐range symmetry breaking is suppressed.

The field‐modified dielectric response of the lattice during this process can be captured via the Lyddane‐Sachs‐Teller relation:

(5) $$\varepsilon \left(\omega \right)=\varepsilon_{\infty }+\sum_{i}\frac{\Delta \varepsilon_{i}\omega_{TOi}^{2}}{\omega_{TOi}^{2}-\omega^{2}-2i\omega_{TOi}\Gamma_{TOi}\left(\omega \right)}$$

Here, ε(ω) is the complex frequency‐dependent permittivity, ε_∞_ is the high‐frequency (electronic) dielectric constant, ω_*TOi*_ is the transverse optical phonon frequency of mode *i*, Δε_*i*_ is the oscillator strength of that mode, Γ_*TOi*_(ω) is the damping function describing phonon relaxation under field influence.

Although ε(ω) describes the longitudinal dielectric response, it is the TO phonons that enter this formulation because the poles of ε(ω) occur at the transverse phonon resonances. The damping function Γ_*TOi*_(ω), in particular, reflects the phonon lifetimes modified by anharmonic interactions and MW‐induced scattering processes. An increase in Γ_*TOi*_(ω) leads to broader phonon features and weaker lattice coherence, conditions that frustrate the propagation of long‐range cooperative distortions such as the Jahn‐Teller transition.

In contrast to MW treatment, furnace annealing proceeds under homogeneous, equilibrium heating, allowing Jahn‐Teller‐active distortions to align over long distances. Once the tetragonal phase is stabilized, as in the Furnace sample, it remains energetically favorable and structurally locked even under subsequent low‐temperature MW exposure (Furnace+MW), provided the cubic phase is never reentered.

Taken together, these results demonstrate that MW annealing decouples local symmetry‐breaking tendencies from global cooperative distortion, consistent with selective disruption of phonon populations critical to orbital‐lattice coherence. The resulting cubic phase in the MW‐treated sample is locally JT‐active, fully oxidized, and structurally ordered at short range, yet globally frustrated in symmetry. This establishes MW fields as a powerful non‐equilibrium tool for stabilizing symmetry‐frustrated phases and tuning collective lattice phenomena in correlated oxides. While the proposed suppression of distortion‐carrying phonon modes provides a consistent framework for interpreting the observed decoupling between local and long‐range order, direct spectroscopic validation, such as in situ Raman or inelastic neutron scattering under microwave excitation, remains an important objective for future studies.

To consolidate these findings, Table 1 summarizes the key structural, electronic, and spectroscopic features of all six CuFe_2_O_4_ samples. While several samples share the same global symmetry (e.g., cubic), their distinct processing histories result in markedly different local environments, oxidation states, and degrees of orbital‐lattice coherence. The table illustrates how MW annealing can either preserve or erase cooperative Jahn‐Teller distortions depending on when it is applied, and how these effects manifest across complementary techniques such as XRD, PDF, and XPS. This comparative view underscores the central thesis of this work: structural symmetry alone is insufficient to infer cooperative behavior without considering the underlying physical pathway.

Taken together, these findings reinforce the broader conclusion that MW annealing enables a unique regime of structural control, wherein oxidation and local reordering occur without triggering symmetry‐breaking phase transitions. This opens a pathway for stabilizing metastable or symmetry‐frustrated phases that are inaccessible via conventional thermal routes. In CuFe_2_O_4_, the MW‐treated sample represents such a phase: a cubic structure with oxidized, locally ordered, JT‐active Cu^2^^+^ centers that fail to organize cooperatively. In the Nanocrystal sample, cooperative distortion is suppressed by finite size and surface energy; in the MW sample, it is suppressed by field‐driven interference with the energetics and kinetics of long‐range ordering.

This ability to decouple local symmetry‐breaking tendencies from global phase transitions has important implications. It suggests that MW fields can be used not merely as a fast or efficient annealing technique but as a tunable control parameter for accessing novel lattice states. In systems where functional properties are tightly coupled to symmetry, such as multiferroics, orbitally ordered materials, or complex spinel oxides, this strategy could be harnessed to engineer materials with hybrid or frustrated structural motifs and possibly new functionalities.

### 4.7 Thermal Stability and Reversibility of the MW‐Stabilized Cubic Phase

**FIGURE 10:**
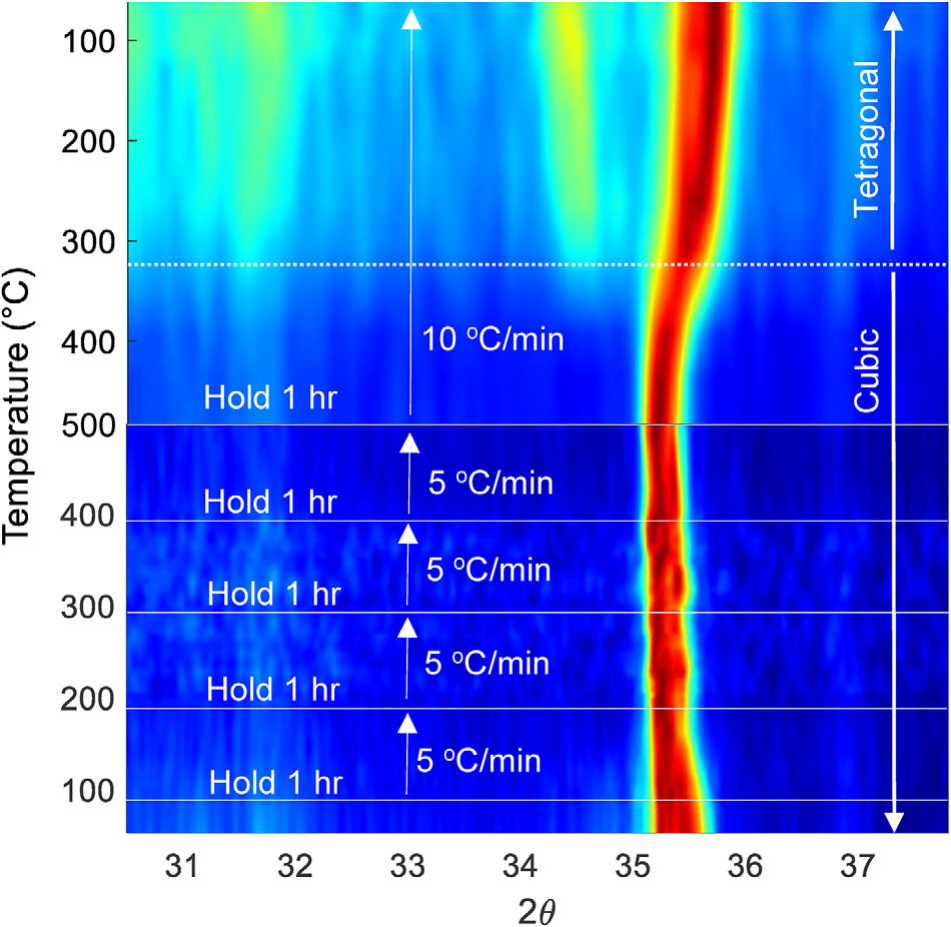
In situ temperature‐dependent 2D XRD map of the Furnace+MW 700°C sample collected during a full heating‐cooling cycle. The sample was heated from room temperature to 500°C at 5°C min^−1^ with 1 h isothermal holds at 100°C, 200°C, 300°C, 400°C, and 500°C, followed by continuous cooling to room temperature at 10°C min^−1^. The sample remains cubic throughout the entire heating cycle, including all hold periods, with no observable tetragonal peak splitting. During cooling, tetragonal splitting emerges near ∼320°C, indicating recovery of long‐range cooperative Jahn‐Teller ordering upon thermal relaxation.

To further examine the stability and reversibility of the MW‐stabilized cubic phase, in situ temperature‐dependent 2D XRD measurements were performed on the Furnace+MW 700°C sample during a complete heating‐cooling cycle. The sample was heated continuously from room‐temperature to 500°C at 5°C min^−^^1^, with intermediate isothermal holds of 1 h at 100°C, 200°C, 300°C, 400°C, and 500°C. After reaching 500°C, the sample was cooled continuously to room‐temperature at 10°C min^−^^1^. The resulting 2D XRD heatmap is shown in Figure 10.

The diffraction data show that the sample remains in the cubic phase throughout the entire heating cycle, including during all intermediate temperature hold periods. No tetragonal peak splitting is observed during heating, indicating that the MW‐stabilized cubic state is kinetically robust over experimentally relevant timescales even at elevated temperatures approaching the known cubic‐to‐tetragonal transition regime of CuFe_2_O_4_. However, during cooling, the onset of tetragonal splitting emerges near ∼320°C, indicating re‐establishment of long‐range cooperative Jahn‐Teller ordering upon thermal relaxation.

This asymmetric heating‐cooling behavior provides important insight into the metastable nature of the MW‐stabilized phase. The persistence of cubic symmetry during prolonged heating demonstrates that the field‐frustrated state is not simply a transient quenched structure that immediately relaxes upon thermal activation. Instead, the results suggest that the establishment of cooperative orbital‐lattice coherence occurs preferentially during cooling, where collective distortion pathways can nucleate and propagate. The delayed recovery of tetragonal symmetry during cooling is therefore consistent with the proposed suppression of long‐range cooperative ordering under prior MW field exposure.

## 5 Conclusion

This study demonstrates that microwave annealing fundamentally alters the structural and electronic evolution of CuFe_2_O_4_ by suppressing the development of cooperative Jahn‐Teller distortions. Using a combination of X‐ray diffraction, pair distribution function analysis, and X‐ray photoelectron spectroscopy, we show that MW treatment leads to complete oxidation to Cu^2+^ and restoration of a distinct short‐range oxygen‐related correlation, conditions that, under conventional thermal processing, would typically result in a tetragonal phase stabilized by CJT distortion. Yet, MW‐treated samples remain globally cubic, revealing a unique non‐equilibrium mechanism that decouples local symmetry breaking from long‐range orbital‐lattice coherence.

Crucially, we find that not all cubic phases are electronically or structurally equivalent. By comparing three cubic samples, namely, Nanocrystal (finite‐size‐limited), MW 700°C (field‐stabilized), and Furnace+MW 700°C (CJT‐relaxed under field exposure), we uncover distinct Cu 2p satellite intensities and binding energies, despite similar oxidation states and average crystallite sizes. These spectroscopic differences reflect variations in Cu‐ligand orbital hybridization and surface coordination, governed by the pathway through which each sample attains its cubic symmetry. The MW 700°C sample retains strong satellite intensity and low binding energy, consistent with JT‐active Cu^2+^ in a structurally relaxed environment. In contrast, the Furnace+MW 700°C sample, which initially exhibits CJT ordering before being relaxed by MW exposure, shows reduced satellite intensity and a more electronically decoupled Cu environment.

We propose that these outcomes stem from MW‐induced suppression of distortion‐carrying phonon modes. Through inelastic interactions with thermally broadened phonon states, we propose that MW‐driven phonon scattering processes may selectively disrupt or depopulate vibrational modes required to propagate cooperative lattice distortions. During slow field‐assisted cooling, this phonon depletion persists, kinetically trapping the system in a metastable cubic state that retains local distortions but lacks global orbital‐lattice coherence. The resulting phases are structurally cubic yet electronically distinct, revealing how non‐equilibrium processing can generate novel structural‐electronic states in correlated oxides.

These findings advance our understanding of how MW fields act not merely as thermal stimuli but as field‐based perturbations that reshape the energy landscape of symmetry‐breaking transitions. By selectively promoting local reordering while frustrating long‐range cooperative instabilities, MW annealing offers a powerful tool to access symmetry‐frustrated or metastable states that lie outside conventional phase diagrams. This strategy may prove broadly applicable to complex oxides where properties such as orbital ordering, spin‐phonon coupling, or correlated phase competition are governed by the fragile balance between local instability and global coherence.

## Author Contributions

**Daryoosh Vashaee**: conceptualization, investigation, funding acquisition, writing – original draft, methodology, validation, visualization, software, formal analysis, project administration, data curation, supervision, resources. **Kelvin Dsouza**: conceptualization, investigation, writing – review and editing, visualization, validation, methodology, software, formal analysis, data curation.

## Conflicts of Interest

The authors declare no conflicts of interest.

## Supporting information




**Supporting File**: advs76001‐sup‐0001‐SuppMat.docx.

## Data Availability

The data that support the findings of this study are available from the corresponding author upon reasonable request.

## Endnote

1 This equipment was developed through AFOSR DURIP support under award number FA9550‐17‐2226 for the project titled *“Field‐induced Chemical and Microstructure Evolution of Dielectric Materials.”*
