# Supplementary material for: Field‐Frustrated Cooperative Distortions: Suppressing Jahn‐Teller Ordering via Microwave Annealing
Source: Adv Sci (Weinh). 2026 Jun 4:e76001. Online ahead of print. doi: 10.1002/advs.76001 (PMC13336883; doi:10.1002/advs.76001)
Supplement: Supplementary file 1 — Supporting File: advs76001‐sup‐0001‐SuppMat.docx. [file ADVS-9999-e76001-s001.docx]

**Field-Frustrated Cooperative Distortions: Suppressing Jahn-Teller Ordering via Microwave Annealing**

Daryoosh Vashaee^1,2^ and Kelvin Dsouza^1^

1Electrical and Computer Engineering Department, North Carolina State University, Raleigh, North Carolina 27616, USA

2Materials Science and Engineering Department, North Carolina State University, Raleigh, North Carolina

Nanocrystal

MW 200 ^o^C

Furnace 700 °C

Furnace + MW 200°C

MW 700 ^o^C

Furnace + MW 700°C

Fe2p

O 1s

Nanocrystal

MW 200 ^o^C (cubic)

Furnace 700 °C

Furnace + MW 200°C

MW 700 ^o^C

Furnace + MW 700°C

Nanocrystal

MW 200 ^o^C (cubic)

Furnace 700 °C

Furnace + MW 200°C

MW 700 ^o^C

Furnace + MW 700°C

C 1s

Figure S1: X-ray photoelectron spectra of Fe 2p (top), O 1s (middle), and C 1s (bottom) core levels for the four CuFe_2_O_4_ samples: Nanocrystal (initial), MW 200 °C, MW 700 °C, Furnace (annealed at 700 °C), Furnace+MW 200 °C, and Furnace+MW 700 °C.

20

30

40

50

60

70

80

90

(

Furnace + MW 700°C (cubic)

Furnace + MW 200°C (tetragonal)

Furnace 700 °C (tetragonal)

Nanocrystal (cubic)

MW 200 C (cubic)

MW 700 ^o^C (cubic)

$$2\theta(^{o})$$

400

311

440

Figure S2: Full-range X-ray diffraction patterns of the CuFe_2_O_4_ samples under different processing conditions: Nanocrystal (as received), MW 200 °C, MW 700 °C, Furnace 700 °C, Furnace+MW 200 °C, and Furnace+MW 700 °C. The extended 2θ range includes higher-angle reflections not shown in the main text (Figure 2) and confirms the phase assignments discussed in the manuscript. The Furnace and Furnace+MW 200 °C samples exhibit peak splitting, especially 311, 400, 440 reflections, consistent with the tetragonal phase, whereas the Nanocrystal, MW-treated, and Furnace+MW 700 °C samples retain cubic symmetry.

**PDF Refinement of Local Structure**

Pair distribution function refinements were carried out using PDFgui/PDFfit2 package^[[1]](#endnote-1)^ to compare the local structural models for the Nanocrystal, Furnace 700 °C, and MW 700 °C CuFe_2_O_4_ samples. For each sample, the experimental PDF, calculated PDF, and residual difference curve are shown in Figures S3-S6. The experimental PDF represents the measured real-space atomic correlations, while the calculated PDF is generated from the refined structural model. The residual curve corresponds to the difference between the experimental and calculated PDFs and provides a measure of the quality of the fit.

The purpose of these refinements was to evaluate whether the short-range PDF features, particularly the MW-specific feature near ~2.5-2.6 Å, can be reproduced by cubic or tetragonal spinel-based structural models. The main PDF peaks arise from Cu-O, Fe-O, O-O, Cu-Fe, Fe-Fe, and Cu-Cu pair correlations. Changes in peak position reflect changes in interatomic distances, while changes in peak width and intensity reflect local disorder, finite coherent domain size, atomic displacement parameters, and local structural distortion.

The refined parameters are summarized in Table S1. The Nanocrystal sample is well described by a nearly cubic spinel model, with a = b = c = 8.31709 Å and an atomic displacement parameter of U = 0.00420 Å^2^. The Furnace sample refines to a tetragonal structure with a = b = 5.834 Å and c = 8.66019 Å, consistent with the XRD evidence for CJT-driven tetragonal distortion. The MW-treated sample requires a more distorted local model, with unequal refined lattice parameters a = 8.415 Å, b = 8.093 Å, and c = 8.528 Å, together with a larger displacement parameter of U = 0.0086 Å^2^. This larger displacement parameter is consistent with a broader distribution of local oxygen environments and field-induced local structural rearrangement.

To further examine the origin of the MW-specific short-range feature, an additional pair-correlation analysis was performed by modifying the oxygen positions in the CuFe_2_O_4_ structure. Oxygen atoms were displaced using a least-squares approach using the lattice-dynamics functionality implemented in CrystalMaker^®^,^[[2]](#endnote-2),^^[[3]](#endnote-3)^ while minimizing changes in the Cu-O and Fe-O bond distances. The resulting model was used to compare the experimental MW PDF with the expected pair-correlation positions for O-O, Cu-O, Fe-O, Cu-Cu, and Fe-Fe pairs. This analysis shows that the ~2.5-2.6 Å feature is most sensitive to oxygen-position rearrangement and is consistent with an O-O/polyhedral correlation associated with local oxygen displacement rather than a simple metal-metal distance. These refinements support the interpretation that MW processing modifies local oxygen-polyhedral coordination while suppressing long-range CJT ordering.

Table S1. Refined structural parameters obtained from PDFgui fits for the Nanocrystal, Furnace 700 °C, and MW 700 °C CuFe_2_O_4_ samples. The Furnace sample refines to tetragonal symmetry, consistent with CJT distortion, while the MW-treated sample shows unequal local lattice parameters and a larger displacement parameter, indicating local structural distortion and oxygen-polyhedral rearrangement.

|  | Nano | Furnace | MW |
| --- | --- | --- | --- |
| Lattice Parameters (Å) | a=b=c=8.31709 | a=b= 5.834 c=8.66019 | a=8.415 b= 8.093 c=8.528 |
| Thermal Parameter (Å^2^) (U_11_,U_22_,U_33_) | 0.00420 | 0.0056 | 0.0086 |

Figure S3. PDFgui refinement of the Nanocrystal CuFe_2_O_4_ sample. The experimental PDF, calculated PDF, and residual difference curve are shown. The fit is consistent with a nearly cubic spinel local structure.

Figure S4. PDFgui refinement of the Furnace 700 °C CuFe_2_O_4_ sample. The refined model shows tetragonal distortion, consistent with the XRD-observed CJT phase.

Figure S5. PDFgui refinement of the MW 700 °C CuFe_2_O_4_ sample. The fit indicates a more distorted local structure with a larger displacement parameter, consistent with MW-induced local oxygen-polyhedral rearrangement.

Figure S6. Pair-correlation analysis of the MW 700 °C CuFe_2_O_4_ sample after oxygen-position modification guided by vibrational-mode displacements. The experimental PDF is shown together with calculated interatomic pair positions for O-O, Cu-O, Fe-O, Cu-Cu, and Fe-Fe correlations. The MW-specific feature near ~2.5-2.6 Å is most consistent with an oxygen-sensitive O-O/polyhedral correlation.

1. Farrow, C. L., P. Juhas, J. W. Liu, D. Bryndin, E. S. Božin, Jacques Bloch, Th Proffen, and S. J. L. Billinge. "PDFfit2 and PDFgui: computer programs for studying nanostructure in crystals." *Journal of Physics: Condensed Matter* 19, no. 33 (2007): 335219. [↑](#endnote-ref-1)
2. Palmer, D. C. (2014). CrystalMaker. CrystalMaker Software Ltd., Begbroke, Oxfordshire, England. [↑](#endnote-ref-2)
3. Gao, Min, David C. Palmer, and Martin T. Dove. "A new approach to molecular and lattice simulations with CrystalMaker® 11: M. Gao et al." *MRS Communications* 15, no. 5 (2025): 1007-1016. [↑](#endnote-ref-3)
